# An integrative review of the physical, mental, and socioeconomic benefits of outdoor hiking

**DOI:** 10.3389/fpubh.2025.1700325

**Published:** 2026-01-07

**Authors:** Tianhang Peng, Zike Zhang, Jiayi Zhang, Wanyuan Liang, Xiuqi Tang

**Affiliations:** 1 School of Sports Science, Beijing Sport University, Beijing, China; 2 Hunan Normal University, Changsha, China; 3 University of Science and Technology Beijing, Beijing, China

**Keywords:** outdoor hiking, physical health, mental health, economic benefits, social benefits, sustainable development

## Abstract

In recent years, outdoor hiking has garnered global attention as an effective health promotion activity. This review synthesizes relevant literature from various databases up to October 2025, assessing the physiological, psychological, and socio-economic impacts of outdoor hiking on individuals. The findings indicate that outdoor hiking significantly improves cardiovascular function, reduces the risk of chronic diseases, and enhances immune function. Additionally, it alleviates stress, improves mood, and helps reduce symptoms of depression. Furthermore, hiking contributes to increased social interaction and community cohesion, while also stimulating tourism and related industries. Existing policies and management measures still have limitations. This paper suggests incorporating trail development into national infrastructure planning, promoting “green social prescribing,” and establishing a standardized framework for benefit assessment to support evidence-based decision-making. In conclusion, outdoor hiking not only enhances individual physical and mental health but also has a positive impact on economic and social development, necessitating policy support and interdisciplinary collaboration for sustainable health promotion.

## 1 Introduction: discovering the value of outdoor hiking

With the acceleration of globalization and urbanization, sedentary lifestyles, persistent psychological stress, and detachment from natural environments have led to rising trends in chronic diseases (such as cardiovascular disease, obesity, and diabetes) and mental health issues (such as anxiety, depression, and stress disorders) (1–6). These issues pose a significant burden on both individual quality of life and public health systems. In response to these challenges, public health policies have increasingly focused on promoting active lifestyles, particularly through outdoor activities like hiking, to improve physical and mental health. Hiking, with its low entry barriers, flexibility, and rich nature-based experiences, has become a globally popular outdoor activity (7). In this study, hiking is defined as a non-competitive outdoor walking activity conducted in natural environments (such as forests, mountains, and rural trails), typically lasting ≥30 min and characterized by natural exposure, psychological restoration, and social interaction (8–10). Existing studies indicate that hiking not only improves cardiovascular function, weight management, and immune function but also alleviates stress, improves mood, and enhances mental health (9–15). Furthermore, hiking promotes social interactions, strengthens community cohesion, and has positive effects on local economies, particularly in tourism and environmental conservation sectors (9, 10, 16, 17). However, most studies have focused either on physiological or psychological effects, often with small sample sizes or cross-sectional designs, lacking interdisciplinary integration. Research on the economic and social benefits remains fragmented. Knowledge gaps still exist regarding unequal participation opportunities, the sustainability of hiking activities, and policy implementation pathways. Therefore, this review adopts an interdisciplinary approach to systematically assess the multidimensional impacts of hiking on health, economics, and social sustainability, examining mechanisms, policies, and practical applications to provide scientific evidence for public health, urban planning, and tourism (18).

## 2 Methods

This study is an integrative review that synthesizes systematic reviews, meta-analyses, and original research evidence, aiming to evaluate the multidimensional benefits of outdoor hiking in terms of physiological, psychological, and socio-economic outcomes. The methodology follows the structure of an umbrella review, integrating quantitative data from existing meta-analyses for the physiological and psychological aspects, while employing a traditional narrative review approach for the socio-economic section. The research follows the PICO/PECO framework to define the study scope: Population: general adults and specific subgroups (such as older individuals and adolescents); Intervention/Exposure: outdoor hiking activities (including trekking, mountain trail walking, and walking in natural environments, excluding indoor or virtual walking, unless used as a comparison); Comparison: non-participants or groups engaged in usual activities; Outcome: physiological health, psychological health, socio-economic benefits, and factors influencing participation barriers and facilitators. Systematic searches were conducted in the PubMed, Scopus, Web of Science, PsycINFO, and SPORTDiscus databases, with a cutoff date of October 20, 2025, including only English-language literature, supplemented by gray literature and reference tracking. The search strategy was developed by an information expert and reviewed according to PRESS standards (the full search strategy is provided in Supplementary Table S1). Two researchers independently screened the literature and extracted data, managing references using EndNote 20.2.1, and documenting study characteristics, intervention information, and primary outcomes using standardized forms. Double data entry and independent verification ensured accuracy, with discrepancies resolved by a third researcher. The quality of the included studies was assessed using the AMSTAR2 tool (see Supplementary Tables S2–S5) (19). Data synthesis primarily involved narrative analysis, with quantitative summaries for measurable outcomes, covering physiological, psychological, and socio-economic benefits, and identifying factors influencing participation barriers and facilitators. Policy, infrastructure, and health promotion strategies were also summarized. The study adhered to the PRISMA 2020 reporting standards, with the literature screening process and final selection of articles presented in Supplementary Figure S1 and Supplementary Table S6.

## 3 Global development and trends in outdoor hiking

### 3.1 Global participation overview, growth projections, and influencing factors

Outdoor leisure activities, particularly hiking, have gained increasing attention worldwide, with participation showing a strong upward trajectory. However, it is noteworthy that, to date, there is no authoritative institution that has published unified and precise comparative data on outdoor hiking participation rates for 2025 across countries. Nonetheless, statistical data from North America, particularly the United States, provides valuable insights into global hiking trends. Long-term forecasts from organizations such as the U.S. Forest Service predict steady growth in hiking participation from 2010 to 2060, with an expected increase of ~7%−10% (7). This growth trend is largely driven by factors such as population growth, socioeconomic changes, and increased leisure time (7, 20). Although some studies suggest a decline in participation among younger generations in certain outdoor activities, the youth demographic remains a key participant group in hiking (7). For Europe and Asia, despite the lack of detailed forecasting data, there are indications of positive trends in hiking through the growth of adventure tourism, increased national park visitation, and the booming outdoor equipment market, suggesting that hiking is similarly on the rise in these regions (21, 22).

Participation in hiking is influenced by a range of factors, with population demographics, socioeconomic conditions, resource accessibility, and climate change being key variables (7, 23–25). While aging populations may see a decline in participation in high-intensity outdoor activities, hiking, with its low-to-moderate physical demands, offers broad adaptability, allowing participation across different age groups with minimal impact from age-related changes. Socioeconomic factors also play a crucial role, with higher-income individuals typically having greater economic capacity and more leisure time to engage in hiking activities, whereas urbanization and accessibility to natural environments may limit urban residents' opportunities for such engagement. The distribution and quality of public natural resources, such as national parks, forests, and trails, directly affect the promotion of hiking activities. The greater the accessibility to these resources, the higher the level of hiking participation. Furthermore, the growing impact of climate change has altered suitable seasons and geographical areas for hiking, significantly influencing the seasonal distribution and regional participation rates of hiking.

### 3.2 Emerging trends: technological integration and practical transformations

With advancements in technology, the integration of navigation and safety technologies has significantly enhanced the accessibility and safety of hiking. Devices such as smartphones, GPS devices, personal locator beacons (PLBs), and hiking apps (off-line maps, route planning, and elevation tracking) have become essential equipment for hikers (7, 26). These technologies not only reduce the risk of getting lost but also enhance hikers' sense of safety in the outdoors, particularly for beginners, encouraging more individuals to attempt more complex trails. Additionally, the rise of social media, online forums, and video platforms (such as YouTube) has facilitated the rapid dissemination of route guides, equipment reviews, and real-time trail information, fostering the widespread dissemination of hiking culture and the formation of online communities (27).

However, the proliferation of technology has sparked debates regarding the relationship between “technology and nature,” with some hikers expressing concerns that over-reliance on technology might diminish the deep connection between individuals and nature, thus impacting the authentic wilderness experience (26, 27). Therefore, finding a balance between enjoying the conveniences brought by technology and maintaining the traditional spirit of hiking has become an ongoing topic of discussion. Furthermore, technological advancements have also led to the emergence of new outdoor activities, such as “Geocaching,” which incorporates GPS positioning to add an element of gaming and exploration to hiking, attracting more young participants and driving the diversification of the hiking experience (7).

## 4 Physical and mental health benefits of hiking

### 4.1 Physical health benefits

**Figure 1:**
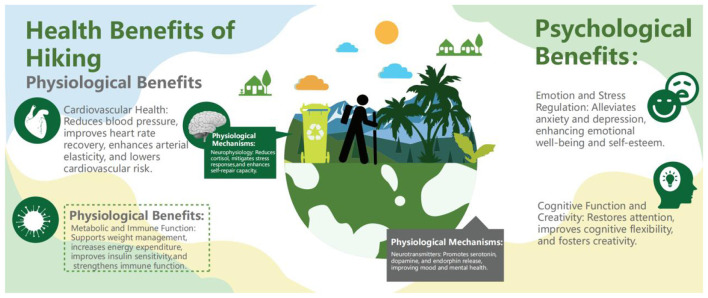
Health benefits of hiking and their potential mechanisms.

Hiking, as a moderate-intensity aerobic exercise, has been widely recognized for its positive effects on physical health (Figure 1). It not only improves cardiovascular health and optimizes metabolic function but also boosts immune system defenses. Numerous studies have shown that hiking significantly helps in the prevention and management of chronic diseases, particularly cardiovascular diseases and type 2 diabetes (28, 29). Furthermore, organizations such as the World Health Organization (WHO) actively promote hiking as an integral part of daily physical activity, recognizing it as an effective means to enhance physical health and delay aging (30–32). Regular hiking activities contribute to weight management, lower blood pressure, improved blood lipid levels, and significantly enhance bone, muscle, and joint health, thereby improving overall quality of life (33–35).

#### 4.1.1 Cardiovascular system improvement

Extensive clinical research supports the positive effects of hiking on cardiovascular health. Hiking has been shown to significantly reduce systolic and diastolic blood pressure in individuals with hypertension, with the benefits being particularly pronounced when hiking in natural environments, such as urban parks or forests (36–41) (Table 1). Regular hiking can also improve heart rate recovery, an indicator of cardiovascular system adaptation and health (42). While hiking may lead to temporary increases in heart rate and blood pressure due to exercise intensity, long-term adherence to hiking helps the body control these fluctuations, thereby reducing the long-term risk of cardiovascular diseases (43, 44). At the molecular level, hiking significantly promotes the expression of proteins related to cholesterol reverse transport (e.g., CD36 and ABCA1) while reducing levels of matrix metalloproteinase-9 (MMP-9), which facilitates vascular remodeling. These molecular changes contribute to lowered blood pressure, improved arterial elasticity, and reduced arterial stiffness, thereby lowering the risk of coronary heart disease and other cardiovascular conditions (38, 45). Additionally, heart rate variability (HRV), a key indicator of autonomic nervous system function, has been shown to significantly improve with hiking in natural environments, highlighting the relaxation and health-promoting effects that nature has on the cardiovascular system (46).

**Table 1:** Summary of meta-analysis results on biomedical and functional health outcomes of hiking.

**First author/Year/Number of included studies**	**Outcome**	***n* (comparisons)**	**Effect size (SDM)**	**95% CI**	***P* value**
Oja P (2018) (40) (*n* = 38 studies)	Body mass	42	−0.134	−0.233, −0.034	< 0.01
	BMI	29	−0.142	−0.257, −0.027	0.015
	Body fat	29	−0.216	−0.336, −0.096	< 0.01
	SBP	35	−0.213	−0.344, −0.082	< 0.01
	DBP	33	−0.166	−0.285, −0.047	< 0.01
	FG	17	−0.211	−0.401, −0.022	0.029
	VO_2_max	31	0.528	0.391, 0.664	< 0.01
Hanson S and Jones A (2015) (41) (*n* = 42 studies)	SBP	440	−3.72	−5.28, −2.17	< 0.01
	DBP	440	−3.14	−4.15, −2.13	< 0.01
	HR	252	−2.88	−4.13, −1.64	< 0.01
	Body fat (%)	328	−1.31	−2.10, −0.52	0.01
	BMI	451	−0.71	−1.19, −0.23	< 0.01
	Total cholesterol (mmol/L)	271	−0.11	−0.22, −0.01	0.03
	VO_2_max	166	2.66	1.67, 3.65	< 0.01
	SF-36 Physical Functioning (points)	68	6.02	0.51, 11.53	0.03
	6-min Walk Distance (m)	65	79.6	53.37, 105.84	< 0.01

The meta-analysis results indicate that outdoor hiking significantly improves key physiological indicators such as body weight, blood pressure, body fat, blood glucose, and aerobic capacity, while also enhancing functional health levels (VO_2_max, 6-min walk distance, SF-36 physical health scores). These effects suggest that hiking not only effectively promotes cardiovascular health and metabolic homeostasis, but also improves overall physical fitness and quality of life, providing evidence-based support for public health interventions. SDM, standardized difference in means; BMI, body mass index; SBP, systolic blood pressure; DBP, diastolic blood pressure; FG, fasting glucose; VO_2_max, maximal oxygen uptake; HR, resting heart rate; TC, total cholesterol.

#### 4.1.2 Metabolic and immune function optimization

Hiking not only benefits weight management and energy balance but also improves metabolic function and enhances immune system defenses. Hiking helps control body weight, increase energy expenditure, improve insulin sensitivity, and reduce the risk of type 2 diabetes and other metabolic diseases (47). Particularly when conducted in natural environments, hiking, such as “Shinrin-yoku” (forest bathing), has been found to modulate the functions of the central nervous system, autonomic nervous system, and endocrine systems, thereby enhancing immune system responses (47). By promoting parasympathetic nervous activity and reducing sympathetic nervous tension, hiking helps the body enter a state of recovery and relaxation, which not only benefits mental health but also boosts immune defenses, preventing chronic diseases (37). In terms of immune function, hiking, especially in natural environments such as forests, has been shown to significantly increase the activity of natural killer (NK) cells, enhancing the body's ability to fight disease (47). Furthermore, hiking reduces stress and regulates stress hormone levels, further enhancing the body's immune response and improving overall health (47).

#### 4.1.3 Exploring the physiological mechanisms of hiking

The health benefits of hiking result from a complex interaction of physiological, psychological, and environmental factors. Physiological evidence shows that forest hiking significantly lowers salivary cortisol levels by modulating the hypothalamic-pituitary-adrenal (HPA) axis, thereby reducing stress responses (48–50). This process enhances parasympathetic nervous activity and reduces sympathetic nervous tension, allowing the body to effectively enter a state of recovery and relaxation, thereby improving self-repair abilities (37, 51). Moreover, hiking also quantifiably demonstrates the effects of this recovery process through physiological indicators such as heart rate variability (HRV). Neuroscientific studies further reveal that hiking significantly reduces the activity of the subgenual prefrontal cortex and amygdala, brain regions associated with negative thinking, suggesting that hiking has a direct neurological basis for stress regulation (52, 53). In terms of neurotransmitters, hiking is thought to promote the release of “happy hormones” such as serotonin, dopamine, and endorphins, which improve mood and mental well-being (54, 55). However, some studies suggest that forest walking may reduce dopamine levels in the blood, indicating that the mechanisms of hiking are more complex and involve the rebalancing of neurotransmitter networks (47). As such, future research will need to focus on the specific regulatory effects of outdoor hiking on neurotransmitter dynamics, exploring its complex physiological and psychological mechanisms (56–58).

## 5 Mental health benefits: the therapeutic power of nature

### 5.1 Emotional and stress regulation

**Figure 2:**
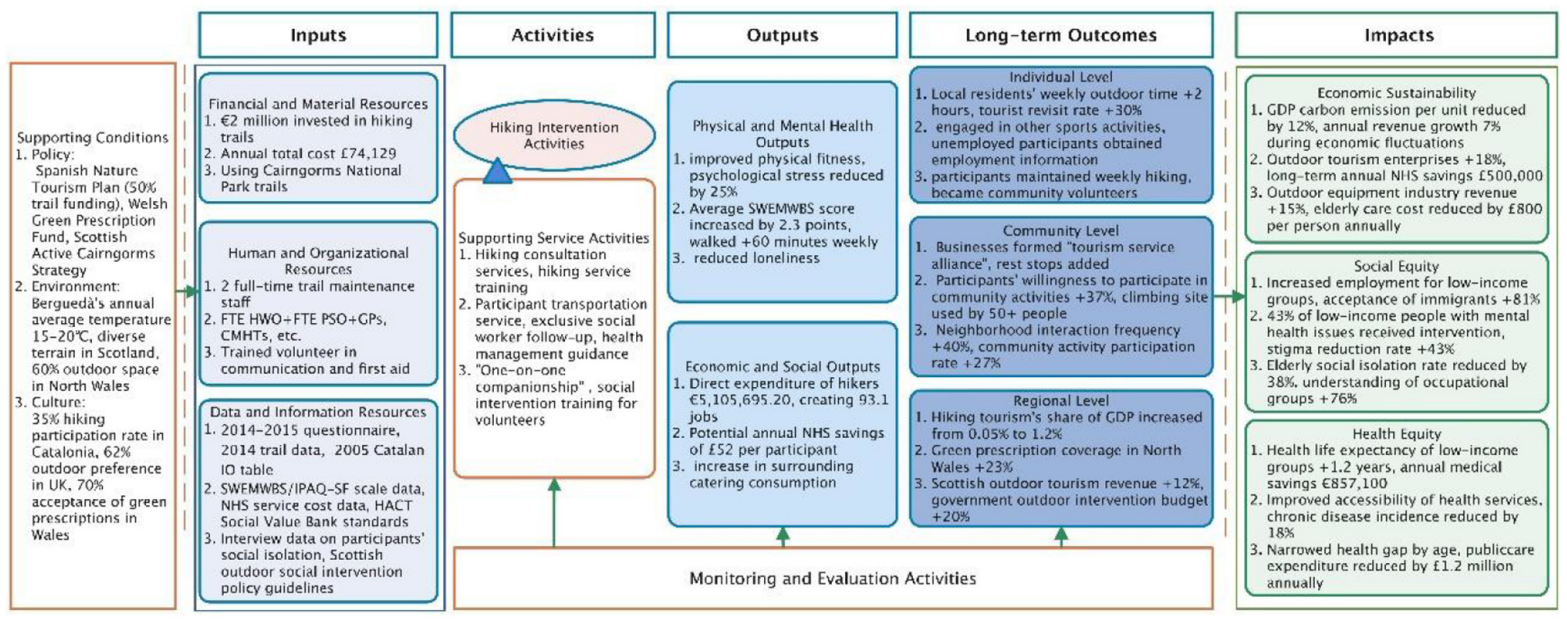
Comprehensive benefits of outdoor hiking interventions: an analysis based on the Theory of Change (ToC) framework.

Multiple systematic reviews and randomized controlled trials (RCTs) have empirically demonstrated that outdoor hiking can alleviate stress, improve mood, enhance self-esteem, and overall well-being, while also promoting psychological recovery and emotional regulation (see Figure 2, Table 2) (41, 59, 60). Natural environments provide quiet, green spaces that facilitate psychological restoration and emotional regulation, which can be explained by theories based on ecological psychology and environmental psychology (56, 61–63). Research indicates that, compared to indoor or urban settings, “green exercise” in natural environments has distinct advantages in improving mood; forest walking (Shinrin-yoku) is particularly effective in reducing anxiety and stress (37, 48, 64), suggesting that natural spaces, through their quiet and green characteristics, play a significant role in promoting psychological health recovery (47, 65, 66).

**Table 2:** Summary of meta-analysis results on mental health outcomes of hiking.

**First Author/Year/Number of Included Studies**	**Outcome**	***n* (comparisons)**	**Effect size (SDM)**	**95% CI**	***P* value**
Grassini S. (2022) (59) (*n* = 7 studies)	Depression (pre- vs. post-nature walk)	175	−0.39	−0.61, −0.18	< 0.01
	Anxiety (pre- vs. post-nature walk)	173	−0.43	−0.69, −0.17	< 0.01
	Depression (nature walk vs. control)	637	−0.23	−0.34, −0.12	< 0.01
	Anxiety (nature walk vs. control)	637	−0.23	−0.87, −0.64	< 0.01
Coventry PA (2021) (60) (*n* = 50 studies)	Depressive mood	1044	−0.64	−1.05, −0.23	< 0.01
	Anxiety	737	−0.94	−1.87, −0.01	< 0.01
	Positive affect	289	0.95	0.59, 1.31	0.101
	Negative affect	347	−0.52	−0.77, −0.26	0.350
Hanson S and Jones A (2015) (41) (*n* = 38 studies)	Depression Score	101	−0.67	−0.97, −0.38	< 0.01

The meta-analysis results indicate that hiking is effective in reducing symptoms of depression and anxiety, enhancing positive emotions, and alleviating negative emotions. SDM, standardized difference in means.

### 5.2 Cognitive function and creativity enhancement

The Attention Restoration Theory (ART) posits that the “soft fascination” of natural environments (e.g., trees, flowing water, birdsong) requires minimal cognitive effort to capture attention, thereby aiding in the restoration of directed attention and enhancing cognitive function and creativity (37, 67, 68). The Stress Reduction Theory (SRT) and Biophilia Hypothesis also emphasize that the evolutionary bond between humans and nature can reduce psychological stress. Hiking interventions, which combine gait rhythm with natural sensory stimulation, have been shown to improve attention, working memory, and cognitive flexibility (69). As a sustainable exercise therapy, hiking has been explored as an adjunctive treatment for mental disorders (e.g., depression, anxiety), with some studies showing that its psychological benefits are comparable to traditional cognitive behavioral therapy or pharmacological interventions (70–72). Additionally, hiking has been found to improve the quality of life, psychological recovery, and social functioning in specific populations (e.g., cancer survivors, veterans) (69, 73–75). However, as of 2025, there is a lack of large-scale, up-to-date meta-analyses specifically focused on “hiking interventions for mental disorders” (73, 74, 76, 77), highlighting the need for high-quality, cross-population longitudinal research to further validate its intervention effects and mechanisms.

### 5.3 Theoretical frameworks and neurophysiological evidence

ART, SRT, and the Biophilia Hypothesis provide psychological mechanisms explaining how natural environments can alleviate mental fatigue and activate the parasympathetic nervous system, thereby reducing stress and anxiety (37, 78). Neurophysiological studies show that forest walking significantly increases parasympathetic activity, reduces sympathetic tension, lowers cortisol levels, and improves blood pressure (79–81). Complex natural landscapes have also been shown to enhance brain executive functions, particularly in cognitive flexibility and perceptual fluency (79, 82).

### 5.4 Quantitative assessment of emotional improvement

In scientific evaluations, standardized scales such as WHO-5, DASS-21, and PANAS are widely used to quantify emotions, stress, and well-being (60, 83–86). Meta-analyses have indicated that exposure to natural environments significantly reduces fatigue (POMS effect size = −0.84), and hiking interventions produce moderate to large effects in improving mood (87–89).

In summary, outdoor hiking, as a moderate-intensity aerobic exercise, derives its psychological health benefits from the interaction between psychological and environmental factors. Empirical evidence supports that hiking improves mood, reduces stress, and enhances cognitive function, while theoretical frameworks (ART, SRT, Biophilia Hypothesis) provide explanations of the psychological and physiological mechanisms involved, offering scientific basis for public health interventions and disease prevention (28, 90, 91).

Physiologically, hiking contributes to cardiovascular health, metabolic regulation, and immune function. Psychologically, it helps alleviate anxiety and depression, enhances emotional health, and improves cognitive function and creativity. These benefits are mediated by neurophysiological mechanisms, including reduced cortisol levels, enhanced self-repair capacity, and the promotion of serotonin, dopamine, and endorphin release.

## 6 Quantitative assessment of economic and social benefits

### 6.1 . Macroeconomic impact: driving regional growth and employment

Hiking tourism, as a significant form of outdoor recreation, has been shown to significantly promote economic growth in regions rich in natural resources, particularly in rural areas. Existing literature categorizes the economic benefits of hiking tourism into three effects: direct effects, indirect effects, and induced effects. The direct effects primarily arise from tourists' expenditures on accommodation, transportation, dining, and equipment. Indirect effects involve the diffusion of hiking-related industry chains, such as equipment manufacturing and the supply of local agricultural products. Induced effects stem from the re-consumption of hiking-related income (17, 92). Empirical studies indicate that these effects can significantly increase regional output and employment. For example, a quantitative study in the Berguedà region of Spain, using input-output models and tourist expenditure surveys, assessed the specific contributions of hiking activities to total output, value-added, and employment (17). Other outdoor recreational activities, such as visits to state parks, non-motorized trail use, rafting, and fishing, also demonstrate similar economic impacts, including job creation, increased local income, and higher regional tax revenues (93–95). Research methodologies primarily include input-output (I-O) models, Keynesian multiplier methods, and tourist expenditure surveys (17, 93, 96, 97). While some studies attempt to explore the relationship between trail mileage and regional GDP or employment rates through regression analysis (98), no standardized quantitative model or official guidelines (such as frameworks from WHO or the UN) currently exist internationally. Therefore, the existing economic benefit data are derived from empirical studies conducted in specific regions, serving as background information to demonstrate the economic potential of hiking tourism. From a macroeconomic perspective, the outdoor recreation industry has become an important component of national economies. For instance, the large scale of the outdoor recreation economy in the United States significantly contributes to GDP (99). This data suggests that outdoor activities, particularly hiking, are not only independent economic phenomena but also key drivers of growth in related industries such as equipment manufacturing, tourism services, and transportation (100).

### 6.2 Health economics perspective: cost-effectiveness and value transformation

The health benefits of hiking can be viewed as a quantifiable economic asset. Existing research has shown that organizing outdoor hiking activities can effectively prevent chronic diseases, improve physical and mental health, and reduce dependence on national healthcare services, thereby saving public healthcare expenditure (e.g., the UK's NHS) (18). Moreover, improvements in physical and mental health can directly translate into increased labor productivity, such as reduced absenteeism and improved efficiency. One study indicated the significant economic loss caused by depression to the U.S. workforce (101), indirectly supporting the potential economic value of outdoor activities like hiking in improving mental health. Health economics research typically uses cost-benefit analysis (CBA) (17) and quality-adjusted life years (QALYs) (102) to monetize health benefits. In this context, CBA quantifies all costs and benefits to assess the social return on investment, while QALYs combine life expectancy and health quality to convert health improvements into economic value.

### 6.3 Contribution to social development: social cohesion and community building

Hiking activities also have significant social impacts, particularly in promoting social cohesion and community building. Research shows that group or partnered hiking can build trust, support, and a sense of belonging through shared challenges, thereby enhancing social capital within the group (103–107). Organized hiking activities, such as community walking groups, have been shown to reduce health inequalities, strengthen neighborhood relationships, and enhance community identity (105, 106). Trust and cooperation gained through social interactions among participants can further promote community entrepreneurship and sustainable development (108). Additionally, specific hiking communities (e.g., long-distance hikers) have formed unique social networks through online and offline activities and information sharing, injecting new vitality into hiking culture (109).

Outdoor hiking interventions not only improve participants' physical and mental health and reduce healthcare costs but also promote community participation, enhance social cohesion, and create economic benefits through tourism and related industries. These multi-level benefits highlight the integrated value of hiking activities in the fields of health economics and social development (Figure 2). The ToC model in Figure 2 is constructed based on the evidence-based ToC framework proposed by WHO, employing a combination of systematic literature mapping, expert consultations, and theoretical deduction. The causal pathways regarding the effects of hiking on cardiovascular, metabolic, and mental health are supported by a wealth of randomized controlled trials and meta-analyses. However, the links between hiking and economic benefits (e.g., employment, GDP growth) as well as social cohesion (e.g., community participation, social capital formation) are, in part, based on theoretical synthesis from interdisciplinary literature, and require further verification through longitudinal and mixed-methods research.

The Theory of Change (ToC) model for outdoor hiking interventions was constructed based on three core references (17, 18, 105). This figure follows the stage-by-stage process outlined in the “Evidence-Informed Theory of Change” published by the World Health Organization (WHO), systematically presenting the outcome chain of outdoor hiking interventions from “Inputs—Activities—Outputs—Long-Term Outcomes—Ultimate Impacts.” On the left side of the figure, the supporting conditions that generate effects (policy, environment, and culture) are listed. The core chain illustrates how hiking activities and associated services can ultimately create comprehensive societal value in the areas of economic sustainability, social equity, and health equity by improving physical and mental health, promoting tourism consumption, and fostering community interaction.

### 6.4 Emerging issues

In recent years, the rise of digital platforms and the sharing economy model has profoundly altered the organization, economic structure, and social dynamics of outdoor hiking activities. Platforms like AllTrails and Meetup have provided hiking enthusiasts with more convenient ways to organize and participate, driving both economic and social transformation in the field. Sharing economy platforms, by connecting service providers (e.g., hiking guides) with consumers, have created new market opportunities. Through this model, individual guides or amateur enthusiasts can bypass traditional intermediaries to provide direct services to tourists, thereby earning more income (110, 111). However, the emergence of digital platforms has also introduced new competitive challenges. By charging commissions and employing price competition strategies, these platforms may influence the income structure of traditional professional guides (112–114). Moreover, the proliferation of platform economies could lead to market profits being concentrated in a few large platform companies, whose algorithms and rule-making processes play a decisive role in the income and market access of guides, raising concerns about fairness (115, 116). From a social inclusivity perspective, digital platforms have a dual impact. On the one hand, they provide marginalized groups (such as people of color, women, and larger-bodied individuals) with more opportunities to participate and platforms for social interaction, thereby increasing their visibility in outdoor activities (117–119). On the other hand, the widespread adoption of these platforms may exacerbate the “digital divide,” particularly for economically disadvantaged groups or those living in rural areas who may lack access to digital devices or related skills, thus hindering their full participation in outdoor activities and exacerbating social inequalities in health behaviors (120, 121).

### 6.5 Challenges and frontiers in evaluation methodology

Although existing evaluation tools and frameworks are becoming more refined, there remain significant challenges in quantifying “non-market values,” especially when it comes to pricing mental health benefits. Methods such as Wellbeing Valuation and Social Return on Investment (SROI) provide directions for addressing this issue (18, 122), but they often rely on complex surveys and statistical models, and the universality and robustness of their results still require further empirical validation (123, 124). Additionally, the standardization of data collection remains a major challenge in evaluations. Current studies predominantly collect data through surveys and interviews (125), yet there is a lack of unified national or international data collection protocols. Establishing standardized data frameworks would improve the transparency and comparability of evaluation results (126–128). While some regions have enhanced the market appeal and tourist satisfaction of trails through certification systems (e.g., Europe's “Leading Quality Trails”) (129), the integration of these certification standards with economic impact assessment methods is still not widespread.

## 7 Major issues and challenges currently faced

With the rapid growth of outdoor hiking, the health, economic, and social benefits it brings are becoming increasingly evident. However, existing research and empirical case studies indicate that the widespread adoption of this activity also exposes a range of challenges, including those related to individual health and safety, social equity, environmental sustainability, and inadequate data and policy support.

Firstly, concerning individual health and safety, although hiking has been shown to effectively improve physical health, beginners and participants in high-risk environments still face significant risks. These include acute illnesses caused by environmental conditions and weather, accidents such as falls or slips, musculoskeletal injuries, and psychological stress (120, 130–136). Evidence suggests that systematic risk education, pre-hike planning, physical conditioning, first aid training, the provision of professional equipment, and the establishment of rescue systems can effectively reduce the incidence of such accidents (132, 133, 135, 137, 138).

Secondly, social inequality and resource distribution issues remain prominent in hiking activities. Low-income communities, ethnic minorities, and people with disabilities encounter significant barriers to access to trail resources, information, and the economic costs associated with hiking (106, 118, 139–142). Empirical research shows that even with interventions such as community walking programs and free walking events like Parkrun, participation inequalities have not been fully eliminated (139, 140, 143–147). Additionally, the rapid growth of hiking activities exerts pressure on vulnerable ecosystems, leading to vegetation destruction, soil compaction, wildlife disturbance, and environmental pollution (144, 148–155). This growth may also negatively impact residents' quality of life and visitors' experiences (156–159). While measures such as visitor flow management, the promotion of “Leave No Trace” principles, and trail maintenance have been implemented, challenges related to funding, staffing, and visitor compliance remain constraints (153, 160).

Lastly, the issues of data collection and policy lag further hinder the sustainable development of outdoor hiking. Current assessments of economic, health, and social benefits suffer from significant methodological differences, inconsistencies in scope, and lack of standardization (17, 97, 161–164), resulting in a delay in policy development relative to the growth of the activity. This is particularly evident in the challenge of balancing environmental protection, visitor experience, community interests, and economic development (160, 165).

## 8 Policy implications, research limitations and future directions

### 8.1 . Policy recommendations

Based on the aforementioned empirical findings, several policy suggestions can be made to enhance the comprehensive benefits of outdoor hiking, while distinguishing between evidence-based recommendations and interpretive inferences. Firstly, the construction of high-quality, accessible trail networks should be prioritized as a key infrastructure investment. Previous studies indicate that trail accessibility and quality are directly related to participation rates, public health improvements, and local economic growth (18). Therefore, trail planning should encompass both urban and rural areas, ensuring barrier-free access, with a particular focus on accessibility for people with disabilities, to maximize social and health benefits. Secondly, interdepartmental collaboration among sectors such as healthcare, environmental protection, sports, and tourism should be promoted. Health economics research demonstrates that outdoor hiking activities improve mental health, alleviate symptoms of depression, and reduce healthcare costs (101, 102). Consequently, at the policy level, hiking could be incorporated into a “green social prescription” system, allowing doctors to prescribe outdoor activities as preventive and rehabilitative interventions. Thirdly, it is crucial to establish a standardized framework for evaluating the benefits of outdoor activities and to improve data collection systems. Existing literature shows significant discrepancies in the methodologies, indicators, and scope of quantifying economic, health, and social benefits (161–164), which hampers the scientific rigor and practicality of policy-making. Developing unified data collection methods, core evaluation indicators, and recommended assessment approaches would provide reliable support for policy decisions and improve performance management. Fourthly, targeted interventions are needed to address social equity and the participation of disadvantaged groups (partially evidence-based and partially interpretive suggestions). Studies indicate that low-income groups, ethnic minorities, and individuals with disabilities face barriers in accessing trail resources (139–141). Policies can improve resource accessibility through community empowerment projects, digital technology, and social platforms, while ensuring equitable participation. Finally, environmental protection and visitor management should be strengthened. Empirical research indicates that hiking activities exert pressure on ecosystems, while visitor flow management, trail maintenance, and the promotion of “Leave No Trace” principles can mitigate environmental degradation (144, 160). Policies should leverage big data and monitoring technologies to optimize visitor distribution, ensuring a safe and sustainable outdoor experience.

### 8.2 . Research limitations and future directions

While existing literature broadly supports the positive effects of hiking on physical and mental health, there are still notable gaps in both methodology and evidence. Firstly, many studies combine hiking with broader categories such as “outdoor activities” or “nature exposure,” making it difficult to isolate the specific effects of hiking (166–168). Secondly, small sample sizes and the predominance of cross-sectional designs limit the generalizability and causal inference of the results. Moreover, most studies rely on self-reported questionnaires, lacking long-term follow-up and objective physiological measures (28, 60, 169, 170). Differences in environmental types (e.g., forests, coastlines, mountains), intervention intensity, and cultural contexts further increase heterogeneity in results (171). Quantitative studies on the dose-response relationship of hiking (including frequency, duration, and intensity) remain limited, and no standardized recommendations for policy or clinical implementation have been established (172). In terms of economic and social dimensions, most research focuses on descriptive analysis, with insufficient cross-regional comparability (96, 173). Additionally, longitudinal studies on the impact of hiking on socio-economic outcomes are almost non-existent (17, 174, 175). Although some studies examine differences in outdoor activity participation among low-income populations (176), the long-term economic impact of hiking as a path to social capital and career development (e.g., guiding or hospitality businesses) has not been explored in depth. Furthermore, there is a lack of research systematically assessing the long-term contributions of hiking activities in reducing chronic disease burden and saving healthcare costs from a macro health economics perspective (177).

To improve the scientific rigor and practicality of outdoor hiking research and policy-making, future studies should adopt a combination of longitudinal designs and mixed methods, incorporating objective physiological indicators, psychological assessments, and economic modeling to build a robust evidence base (56, 123, 124). Furthermore, there is a need to explore the differential responses of various groups (e.g., children, older individuals, and those with specific health conditions) to intervention dose, frequency, and intensity, providing a foundation for personalized interventions and policy optimization. Additionally, interdisciplinary collaboration—encompassing public health, economics, and geographic information sciences—is essential for establishing a standardized framework for evaluating the health, economic, and social benefits of hiking. This would aid in the scientific evaluation of policy effectiveness and guide resource allocation, thereby achieving the integrated enhancement of social equity, environmental sustainability, and public health goals. Moreover, the use of digital technologies and data analytics could facilitate dynamic management, optimize visitor flow distribution, and reduce ecological pressure. This would maximize the health, social, and economic benefits of outdoor hiking activities while ensuring participant safety and environmental sustainability, providing quantifiable scientific support for policy decisions (18, 144, 150).

## 9 Conclusion

This study demonstrates that outdoor hiking activities have widespread and profound effects on enhancing individual physical and mental health, fostering social integration, and promoting local economic development. Specifically, outdoor hiking not only reduces the incidence of chronic diseases and promotes mental health improvements but also generates significant economic benefits, especially in tourism, employment, and related industries. However, with the increasing participation in hiking, new challenges such as resource inequality, environmental burdens, and personal safety risks have emerged. Therefore, ensuring the sustainable development of outdoor hiking requires systematic and comprehensive policy interventions and management measures. Policymakers should prioritize addressing resource allocation inequalities, enhancing infrastructure, optimizing trail networks, and promoting public health through initiatives like green social prescribing. Meanwhile, future research should focus on long-term health benefits, particularly the economic quantification of mental health improvements, and explore the effectiveness of personalized intervention strategies. These efforts will lay a solid foundation for promoting the sustainable development of outdoor hiking and ensuring its widespread benefits across society. Therefore, further strengthening interdisciplinary collaboration and policy support will be key to maximizing the health, economic, and social benefits of outdoor hiking activities.

## References

[1] Romain AJ Marleau J Baillot A. Association between physical multimorbidity, body mass index and mental health/disorders in a representative sample of people with obesity. J Epidemiol Commun Health. (2019) 73:874–80. doi: 10.1136/jech-2018-211497 31201257

[2] Lavallee KL Zhang XC Schneider S Margraf J. Obesity and mental health: a longitudinal, cross-cultural examination in Germany and China. Front Psychol. (2021) 12:712567. doi: 10.3389/fpsyg.2021.712567 34646201 PMC8504480

[3] Samudra RP Heboyan V. Examining the connection between health outcomes, state political ideology, and food access in the United States. J Public Health Manag Pract JPHMP. (2023) 29:E284–92. doi: 10.1097/PHH.0000000000001786 37536664

[4] Geiger S Steinbach J Skoda EM Jahre L Rentrop V Kocol D . Needs and demands for e-mental health interventions in individuals with overweight and obesity: user-centred design approach. Obes Facts. (2023) 16:173–83. doi: 10.1159/000527914 36442465 PMC10028369

[5] Muro A Mateo C Parrado E Subirana-Malaret M Moya M Garriga A . Forest bathing and hiking benefits for mental health during the COVID-19 pandemic in Mediterranean regions. Eur J For Res. (2023) 142:415–26. doi: 10.1007/s10342-023-01531-6 36779181 PMC9896453

[6] Teixeira A Gabriel R Quaresma L Alencoão A Martinho J Moreira H. Obesity and natural spaces in adults and older people: a systematic review. J Phys Act Health. (2021) 18:714–27. doi: 10.1123/jpah.2020-0589 33883287

[7] Cordell HK. Outdoor Recreation Trends and Futures: A Technical Document Supporting the Forest Service 2010 RPA Assessment. Asheville, NC: US Department of Agriculture Forest Service, Southern Research Station (2012). p. 1–167. doi: 10.2737/SRS-GTR-150

[8] Fattorini L Pittiglio G Federico B Pallicca A Bernardi M Rodio A. Workload comparison between hiking and indoor physical activity. J Strength Cond Res. (2012) 26:2883. doi: 10.1519/JSC.0b013e318242a61e 22158090

[9] Acevedo-Duque Á Llanos-Herrera GR García-Salirrosas EE Simón-Isidoro S Álvarez-Herranz AP Álvarez-Becerra R . Scientometric analysis of hiking tourism and its relevance for wellbeing and knowledge management. Int J Environ Res Public Health. (2022) 19:8534. doi: 10.3390/ijerph19148534 35886386 PMC9319550

[10] Mitten D Overholt JR Haynes FI D'Amore CC Ady JC. Hiking: a low-cost, accessible intervention to promote health benefits. Am J Lifestyle Med. (2018) 12:302–10. doi: 10.1177/1559827616658229 32063815 PMC6993091

[11] Liao X Zhu Y Lu L Li W Zhang L Ji C . Maternal manganese activates anti-apoptotic-related gene expressions via miR-1551 and miR-34c in embryonic hearts from maternal heat stress (Gallus gallus). J Therm Biol. (2019) 84:190–9. doi: 10.1016/j.jtherbio.2019.07.014 31466753

[12] Lesser IA Thomson CJ. A pre-post study design exploring the potential benefits of a hiking intervention for active and inactive older adults. J Aging Phys Act. (2025) 33:17–26. doi: 10.1123/japa.2023-0347 39151906

[13] Niedermeier M Einwanger J Hartl A Kopp M. Affective responses in mountain hiking-a randomized crossover trial focusing on differences between indoor and outdoor activity. PLoS ONE. (2017) 12:e0177719. doi: 10.1371/journal.pone.0177719 28520774 PMC5433751

[14] Xu P Huang Y Hou Q Cheng J Ren Z Ye R . Relationship between physical activity and mental health in a national representative cross-section study: its variations according to obesity and comorbidity. J Affect Disord. (2022) 308:484–93. doi: 10.1016/j.jad.2022.04.037 35439463

[15] Bettmann JE Speelman E Blumenthal E Couch S McArthur T. How does nature exposure affect adults with symptoms of mental illness? A meta-analysis. Int J Mental Health Nurs. (2024) 33:1889–907. doi: 10.1111/inm.13400 39209768

[16] Smiley A Ramos W Elliott L Wolter S. Comparing the trail users with trail non-users on physical activity, sleep, mood and well-being index. Int J Environ Res Public Health. (2020) 17:6225. doi: 10.3390/ijerph17176225 32867170 PMC7503490

[17] Raya JM Martinez-Garcia E Celma D. Economic and social yield of investing in hiking tourism: the case of Berguedà, Spain. J Travel Tour Mark. (2017) 35:1–14. doi: 10.1080/10548408.2017.1350252

[18] Makanjuola A Lynch M Hartfiel N Cuthbert A Edwards RT. Prevention of poor physical and mental health through the green social prescribing opening doors to the outdoors programme: a social return on investment analysis. Int J Environ Res Public Health. (2023) 20:6111. doi: 10.3390/ijerph20126111 37372698 PMC10298668

[19] Shea BJ Reeves BC Wells G Thuku M Hamel C Moran J . AMSTAR 2: a critical appraisal tool for systematic reviews that include randomised or non-randomised studies of healthcare interventions, or both. BMJ. (2017) 358:j4008. doi: 10.1136/bmj.j4008 28935701 PMC5833365

[20] Marafa LM Ting HY Cheong CK. Perceived benefits of hiking as an outdoor recreation activity in Hong Kong. LICERE Rev Programa Pós-grad Interdiscip Estud Lazer. (2007) 10. doi: 10.35699/1981-3171.2007.934

[21] Pal K. A review of employee engagement and strategies implementation in virtual work environment. Int J Sci Res Sci Eng Technol. (2021) 48:114–21. doi: 10.32628/IJSRSET218425

[22] Dang TT Antolin P Oxley H. Fiscal Implication of Ageing: Projections of Age-Related Spending. Rochester, NY: Social Science Research Network (2001). Available online at: https://papers.ssrn.com/abstract=607122. doi: 10.2139/ssrn.607122

[23] Gramann JH. Ethnicity, Race, and Outdoor Recreation. A Review of Trends, Policy, and Research. Vicksburg, MS: U.S. Army Corps of Engineers, Waterways Experiment Station (2025). Available online at: https://apps.dtic.mil/sti/html/tr/ADA306913/

[24] White EM Bowker JM Askew AE Langner LL Arnold JR English DBK. Federal Outdoor Recreation Trends: Effects on Economic Opportunities. Portland, OR: US Department of Agriculture, Pacific Northwest Research Station (2016). doi: 10.2737/PNW-GTR-945

[25] Askew AE Bowker JM. Impacts of climate change on outdoor recreation participation: outlook to 2060. J Park Recreat Adm. (2018) 36:97–120. doi: 10.18666/JPRA-2018-V36-I2-8316

[26] Hyatt E Pointon M Harvey M Innocenti P. Whither wilderness? An investigation of technology use by long-distance backpackers. J Assoc Inf Sci Technol. (2021) 72:683–98. doi: 10.1002/asi.24437

[27] Fields SG. Technology on the Trail: Using Cultural Probes to Understand Hikers. Blacksburg: Virginia Tech (2017). Available online at: http://hdl.handle.net/10919/78726.

[28] Huber D Freidl J Pichler C Bischof M Kiem M Weisböck-Erdheim R . Long-term effects of mountain hiking vs. forest therapy on physical and mental health of couples: a randomized controlled trial. Int J Environ Res Public Health. (2023) 20:1469. doi: 10.3390/ijerph20021469 36674227 PMC9859399

[29] Malem R Ristiani R Ali Puteh M. Brisk Walking exercise has benefits of lowering blood pressure in hypertension sufferers: a systematic review and meta-analysis. Iran J Public Health. (2024) 53:774–84. doi: 10.18502/ijph.v53i4.15554 39444461 PMC11493580

[30] Wen CP Wai JP Tsai MK Yang YC Cheng TY Lee MC . Minimum amount of physical activity for reduced mortality and extended life expectancy: a prospective cohort study. Lancet. (2011) 378:1244–53. doi: 10.1016/S0140-6736(11)60749-6 21846575

[31] Smith E Cusack T Cunningham C Blake C. The influence of a cognitive dual task on the gait parameters of healthy older adults: a systematic review and meta-analysis. J Aging Phys Act. (2017) 25:671–86. doi: 10.1123/japa.2016-0265 28253049

[32] Murtagh EM Murphy MH Boone-Heinonen J. Walking: the first steps in cardiovascular disease prevention. Curr Opin Cardiol. (2010) 25:490. doi: 10.1097/HCO.0b013e32833ce972 20625280 PMC3098122

[33] Woodcock J Givoni M Morgan AS. Health impact modelling of active travel visions for England and Wales using an integrated transport and health impact modelling tool (ITHIM). PLoS ONE. (2013) 8:e51462. doi: 10.1371/journal.pone.0051462 23326315 PMC3541403

[34] Kahlmeier S Cavill N Thondoo M Rutter H de Sa TH Racioppi F . The Health Economic Assessment Tool (HEAT) for walking and cycling - experiences from 10 years of application of a health impact assessment tool in policy and practice. Front Sports Active Living. (2023) 5:1146761. doi: 10.3389/fspor.2023.1146761 37389275 PMC10305804

[35] Götschi T Kahlmeier S Castro A Brand C Cavill N Kelly P . Integrated impact assessment of active travel: expanding the scope of the health economic assessment tool (HEAT) for walking and cycling. Int J Environ Res Public Health. (2020) 17:7361. doi: 10.3390/ijerph17207361 33050184 PMC7600508

[36] Papale O Festino E Di Rocco F Foster C Prestanti I Serafini S . The impact of a multidimensional physical activity intervention on glycemic control in type 1 diabetes: a preliminary study. J Funct Morphol Kinesiol. (2025) 10:163. doi: 10.3390/jfmk10020163 40407447 PMC12101377

[37] Moore MS. The interleaving trails of lifestyle and wilderness. Am J Lifestyle Med. (2023) 17:470–5. doi: 10.1177/15598276221140854 37426735 PMC10328209

[38] Webb R Thompson JE Ruffino JS Davies NA Watkeys L Hooper S . Evaluation of cardiovascular risk-lowering health benefits accruing from laboratory-based, community-based and exercise-referral exercise programmes. BMJ Open Sport Exerc Med. (2016) 2:e000089. doi: 10.1136/bmjsem-2015-000089 27900165 PMC5117059

[39] Papale O Festino E Rocco FD Maio MD Cortis C Fusco A . Psychophysiological data harmonization for the sustainability of outdoor activities. Sustainability. (2023) 15:15838. doi: 10.3390/su152215838

[40] Oja P Kelly P Murtagh EM Murphy MH Foster C Titze S. Effects of frequency, intensity, duration and volume of walking interventions on CVD risk factors: a systematic review and meta-regression analysis of randomised controlled trials among inactive healthy adults. Br J Sports Med. (2018) 52:769–75. doi: 10.1136/bjsports-2017-098558 29858464

[41] Hanson S Jones A. Is there evidence that walking groups have health benefits? A systematic review and meta-analysis. Br J Sports Med. (2015) 49:710–5. doi: 10.1136/bjsports-2014-094157 25601182 PMC4453623

[42] Skaliy AV Mulyk KV Ban Z . Assessment of the functional state of the cardiovascular system of students during a mountain hiking trip. Slobozhanskyi Her Sci Sport. (2023) 27: 158–165. doi: 10.15391/snsv.2023-3.007

[43] Mieda R Matsui Y Tobe M Kanamoto M Suto T Saito S. Education program for prevention of outdoor accidents in middle-high aged trekkers: monitoring of change in blood pressure and heart rate during exercise. Prev Med Rep. (2021) 23:101396. doi: 10.1016/j.pmedr.2021.101396 34094816 PMC8164081

[44] Faulkner J Gerhard J Stoner L Lambrick D. Self-paced walking within a diverse topographical environment elicits an appropriate training stimulus for cardiac rehabilitation patients. Rehabil Res Pract. (2012) 2012:140871. doi: 10.1155/2012/140871 22848835 PMC3400393

[45] Manning JW Montes J Stone TM Rietjens RW Young JC DeBeliso M . Cardiovascular and perceived exertion responses to leisure trail hiking. J Outdoor Recreat Educ Leadership. (2015) 7:83–92. doi: 10.18666/JOREL-2015-V7-I2-7005

[46] Martinaitiene D Sampaio F Demetrovics Z Gjoneska B Portačenko J Damulevičiute A . A randomised controlled trial assessing the effects of weather sensitivity profile and walking in nature on the psychophysiological response to stress in individuals with coronary artery disease. A study protocol. BMC Psychol. (2024) 12:82. doi: 10.1186/s40359-024-01574-3 38374158 PMC10877807

[47] López-Pousa S Bassets Pagès G Monserrat-Vila S de Gracia Blanco M Hidalgo Colomé J Garre-Olmo J. Sense of well-being in patients with fibromyalgia: aerobic exercise program in a mature forest-a pilot study. Evid Based Complement Alternat Med. (2015) 2015:614783. doi: 10.1155/2015/614783 26557151 PMC4628674

[48] Shin MJ You JH Kim JU Shin WS. Effects of exercise intensity differences in forest therapy programs on immunoglobulin a and dehydroepiandrosterone levels in older adults. Forests. (2024) 15:577. doi: 10.3390/f15040577

[49] Niedermeier M Grafetstätter C Hartl A Kopp M. A randomized crossover trial on acute stress-related physiological responses to mountain hiking. Int J Environ Res Public Health. (2017) 14:905. doi: 10.3390/ijerph14080905 28800067 PMC5580608

[50] O'mara S. Biopsychosocial functions of human walking and adherence to behaviourally demanding belief systems: a narrative review. Front Psychol. (2021) 12:654122. doi: 10.3389/fpsyg.2021.654122 34421710 PMC8371042

[51] Farrow MR Washburn K. A review of field experiments on the effect of forest bathing on anxiety and heart rate variability. Glob Adv Health Med. (2019) 8:2164956119848654. doi: 10.1177/2164956119848654 31192052 PMC6540467

[52] Mau M Aaby A Klausen SH Roessler KK. Are long-distance walks therapeutic? A systematic scoping review of the conceptualization of long-distance walking and its relation to mental health. Int J Environ Res Public Health. (2021) 18:7741. doi: 10.3390/ijerph18157741 34360035 PMC8345809

[53] Sudimac S Sale V Kühn S. How nature nurtures: amygdala activity decreases as the result of a one-hour walk in nature. Mol Psychiatry. (2022) 27:4446–52. doi: 10.1038/s41380-022-01720-6 36059042 PMC9734043

[54] Wang M Jiang C Huang Y He X Deng L. The association of outdoor walking per week with mental health and costs of psychotropic drugs in adults. J Commun Health. (2023) 48:136–40. doi: 10.1007/s10900-022-01157-6 36318361

[55] Walter KH Otis NP Glassman LH Ray TN Michalewicz-Kragh B Kobayashi Elliott KT . Comparison of surf and hike therapy for active duty service members with major depressive disorder: study protocol for a randomized controlled trial of novel interventions in a naturalistic setting. Contemp Clin Trials Commun. (2019) 16:100435. doi: 10.1016/j.conctc.2019.100435 31485546 PMC6717066

[56] Thompson Coon J Boddy K Stein K Whear R Barton J Depledge MH. Does participating in physical activity in outdoor natural environments have a greater effect on physical and mental wellbeing than physical activity indoors? A systematic review. Environ Sci Technol. (2011) 45:1761–72. doi: 10.1021/es102947t 21291246

[57] Roberts L Jones G Brooks R. Why do you ride?: A characterization of mountain bikers, their engagement methods, and perceived links to mental health and well-being. Front Psychol. (2018) 9:1642. doi: 10.3389/fpsyg.2018.01642 30283372 PMC6156442

[58] Barton J Pretty J. What is the best dose of nature and green exercise for improving mental health? A multi-study analysis. Environ Sci Technol. (2010) 44:3947–55. doi: 10.1021/es903183r 20337470

[59] Grassini S. A Systematic review and meta-analysis of nature walk as an intervention for anxiety and depression. J Clin Med. (2022) 11:1731. doi: 10.3390/jcm11061731 35330055 PMC8953618

[60] Coventry PA Brown JE Pervin J Brabyn S Pateman R Breedvelt J . Nature-based outdoor activities for mental and physical health: systematic review and meta-analysis. SSM Popul Health. (2021) 16:100934. doi: 10.1016/j.ssmph.2021.100934 34646931 PMC8498096

[61] Bratman GN Hamilton JP Hahn KS Daily GC Gross JJ. Nature experience reduces rumination and subgenual prefrontal cortex activation. Proc Nat Acad Sci. (2015) 112:8567–72. doi: 10.1073/pnas.1510459112 26124129 PMC4507237

[62] Piva G Caruso L Gómez AC Calzolari M Visintin EP Davoli P . Effects of forest walking on physical and mental health in elderly populations: a systematic review. Rev Environ Health. (2024) 39:121–36. doi: 10.1515/reveh-2022-0093 36239186

[63] Noseworthy M Peddie L Buckler EJ Park F Pham M Pratt S . The effects of outdoor versus indoor exercise on psychological health, physical health, and physical activity behaviour: a systematic review of longitudinal trials. Int J Environ Res Public Health. (2023) 20:1669. doi: 10.3390/ijerph20031669 36767034 PMC9914639

[64] Park BJ Tsunetsugu Y Kasetani T Kagawa T Miyazaki Y. The physiological effects of Shinrin-yoku (taking in the forest atmosphere or forest bathing): evidence from field experiments in 24 forests across Japan. Environ Health Prev Med. (2010) 15:18–26. doi: 10.1007/s12199-009-0086-9 19568835 PMC2793346

[65] Kelly P Williamson C Niven AG Hunter R Mutrie N Richards J. Walking on sunshine: scoping review of the evidence for walking and mental health. Br J Sports Med. (2018) 52:800–6. doi: 10.1136/bjsports-2017-098827 29858467

[66] Greco G Centrone C Poli L Silva AF Russo L Cataldi S . Impact of coastal walking outdoors and virtual reality indoor walking on heart rate, enjoyment levels and mindfulness experiences in healthy adults. J Func Morphol Kinesiol. (2024) 9:11. doi: 10.3390/jfmk9010011c 38249088 PMC10801470

[67] Niedermeier M Grafetstätter C Kopp M Huber D Mayr M Pichler C . The role of anthropogenic elements in the environment for affective states and cortisol concentration in mountain hiking—a crossover trial. Int J Environ Res Public Health. (2019) 16:290. doi: 10.3390/ijerph16020290 30669640 PMC6352183

[68] Bailey AW Kang HK. Walking and sitting outdoors: which is better for cognitive performance and mental states? Int J Environ Res Public Health. (2022) 19:16638. doi: 10.3390/ijerph192416638 36554519 PMC9778927

[69] Victorson D Doninger G Victorson S Victorson G Hall L Maletich C . Psychosocial and biological outcomes of immersive, mindfulness-based treks in nature for groups of young adults and caregivers affected by cancer: results from a single arm program evaluation from 2016-2021. Int J Environ Res Public Health. (2021) 18:12622. doi: 10.3390/ijerph182312622 34886348 PMC8657001

[70] Kleinstäuber M Reuter M Doll N . Rock climbing and acute emotion regulation in patients with major depressive disorder in the context of a psychological inpatient treatment: a controlled pilot trial. Psychol Res Behav Manag. (2017) 10:277–81. doi: 10.2147/PRBM.S143830 28860880 PMC5566792

[71] Luttenberger K Stelzer EM Först S Schopper M Kornhuber J Book S. Indoor rock climbing (bouldering) as a new treatment for depression: study design of a waitlist-controlled randomized group pilot study and the first results. BMC Psychiatry. (2015) 15:201. doi: 10.1186/s12888-015-0585-8 26302900 PMC4548691

[72] Dorscht L Karg N Book S Graessel E Kornhuber J Luttenberger K. A German climbing study on depression: a bouldering psychotherapeutic group intervention in outpatients compared with state-of-the-art cognitive behavioural group therapy and physical activation – study protocol for a multicentre randomised controlled trial. BMC Psychiatry. (2019) 19:154. doi: 10.1186/s12888-019-2140-5 31101097 PMC6525374

[73] Harmon J Kyle G. Connecting to the trail: natural spaces as places of healing. Leisure Sci. (2022) 44:1112–27. doi: 10.1080/01490400.2020.1712282

[74] Walter KH Otis NP Ray TN Glassman LH Beltran JL Kobayashi Elliott KT . A randomized controlled trial of surf and hike therapy for US active duty service members with major depressive disorder. BMC Psychiatry. (2023) 23:109. doi: 10.1186/s12888-022-04452-7 36805672 PMC9936467

[75] Meloni M Natale D Caria A Porco IG Ventura L Bandiera P . Land- and water-based sports activities in natural environments as a group exercise for Parkinson's disease: proof-of-concept pilot study. Sport Sci Health. (2025) 21:839–51. doi: 10.1007/s11332-024-01321-6

[76] Ma J Lin P Williams J. Effectiveness of nature-based walking interventions in improving mental health in adults: a systematic review. Curr Psychol. (2024) 43:9521–39. doi: 10.1007/s12144-023-05112-z

[77] Rosa C D Chaves T S Collado S . The effect of nature-based adventure interventions on depression: a systematic review. Environ Behav. (2023) 55:140–74. doi: 10.1177/00139165231174615

[78] Park S Kim E Kim G Kim S Choi Y Paek D. What activities in forests are beneficial for human health? A systematic review. Int J Environ Res Public Health. (2022) 19:2692. doi: 10.3390/ijerph19052692 35270397 PMC8909949

[79] Lamatungga KE Pichlerová M Halamová J . Forests serve vulnerable groups in times of crises: improved mental health of older adults by individual forest walking during the COVID-19 pandemic. Front Forests Glob Change. (2024) 7. doi: 10.3389/ffgc.2024.1287266

[80] Lee J Tsunetsugu Y Takayama N . Influence of forest therapy on cardiovascular relaxation in young adults. Evid Based Complement Alternat Med. (2014) 2014:834360. doi: 10.1155/2014/834360 24660018 PMC3934621

[81] Lee DG Lee MM Jeong YM Kim JG Yoon YK Shin WS. Influence of forest visitors' perceived restorativeness on social-psychological stress. Int J Environ Res Public Health. (2021) 18:6328. doi: 10.3390/ijerph18126328 34208025 PMC8296131

[82] Wan R Wan R Qiu Q. Progress and prospects of research on the impact of forest therapy on mental health: a bibliometric analysis. Forests. (2024) 15:1013. doi: 10.3390/f15061013

[83] Mayer K Lukács A. Motivation and mental well-being of long-distance hikers: a quantitative and qualitative approach. Heliyon. (2021) 7:e06960. doi: 10.1016/j.heliyon.2021.e06960 34007934 PMC8111588

[84] Marselle MR Irvine KN Warber SL. Walking for well-being: are group walks in certain types of natural environments better for well-being than group walks in urban environments? Int J Environ Res Public Health. (2013) 10:5603–28. doi: 10.3390/ijerph10115603 24173142 PMC3863862

[85] Wen Y Gu X Deng W Yan Q Pan Y Liu Y . The effects of dynamic and static forest bathing (Shinrin-yoku) on physiological and psychological health in males and females. Forests. (2023) 14:1592. doi: 10.3390/f14081592

[86] Watson D Clark LA Tellegen A. Development and validation of brief measures of positive and negative affect: the PANAS scales. J Pers Soc Psychol. (1988) 54:1063–70. doi: 10.1037//0022-3514.54.6.1063 3397865

[87] Kanchibhotla D Harsora P Gupte P Mehrotra S Sharma P Trehan N. Alleviating work exhaustion, improving professional fulfillment, and influencing positivity among healthcare professionals during COVID-19: a study on Sudarshan Kriya Yoga. Front Psychol. (2022) 13:670227. doi: 10.3389/fpsyg.2022.670227 35910997 PMC9326464

[88] Enrique Roig A Molinari G Miralles I . El Mejor Self Posible: Una intervención dirigida a generar emociones positivas. Res Prelim. (2015) 2:215–23. doi: 10.6035/AgoraSalut.2015.2.21

[89] Song S Tu R Lu Y Yin S Lin H Xiao Y. Restorative Effects from green exposure: a systematic review and meta-analysis of randomized control trials. Int J Environ Res Public Health. (2022) 19:14506. doi: 10.3390/ijerph192114506 36361386 PMC9658851

[90] Bull FC Al-Ansari SS Biddle S Borodulin K Buman MP Cardon G . World Health Organization 2020 guidelines on physical activity and sedentary behaviour. Br J Sports Med. (2020) 54:1451–62. doi: 10.1136/bjsports-2020-102955 33239350 PMC7719906

[91] Stoltzfus KB Naylor D Cattermole T Ankeney A Mount R Chang R . Blood pressure changes while hiking at moderate altitudes: a prospective cohort study. Int J Environ Res Public Health. (2020) 17:7978. doi: 10.3390/ijerph17217978 33142996 PMC7663232

[92] Kastenholz E Rodrigues Á. Discussing the potential benefits of hiking tourism in Portugal. Anatolia. (2007) 18:5–21. doi: 10.1080/13032917.2007.9687033

[93] Nagler AM Bastian CT Taylor DT . Community economic contributions from recreational trails usage on public lands: implications from a comprehensive Wyoming Case Study. West Econ Forum. (2013) 12:1–11.

[94] Bergstrom JC Cordell HK Watson AE Ashley GA. Economic impacts of state parks on state economies in the South. J Agric Appl Econ. (1990) 22:69–77. doi: 10.1017/S1074070800001826

[95] English DBK Bowker JM. Economic impacts of guided whitewater rafting: a study of five rivers. J Am Water Resour Assoc. (2025) 32:1319–28. doi: 10.1111/j.1752-1688.1996.tb03500.x

[96] Lukoseviciute G Pereira LN Panagopoulos T. The economic impact of recreational trails: a systematic literature review. J Ecotour. (2022) 21:366–93. doi: 10.1080/14724049.2022.2030745

[97] Comley V Mackintosh C. The Economic Impact of Outdoor Recreation in the UK: The Evidence. Liverpool: SRA/Liverpool John Moores University location (2014).

[98] Dunlop SR Ukkusuri S Thakkar DJ Mittal S Patil U Gala J . Economic Effect of Active Transportation Features and the Association Between the Healthcare Industry and Transportation. West Lafayette, IN: Purdue University (2023).

[99] Spradlin TJ Givens JE. Framing climate change in local context: newspaper coverage of climate change in three mountain towns in the intermountain west compared to national coverage. Newsp Res J. (2022) 43:073953292211064. doi: 10.1177/07395329221106485

[100] Maples J Bradley M. Outdoor recreation and rural transitions in Central Appalachia: revisiting the economic impact of rock climbing in Kentucky's Red River Gorge. J Econ Impact. (2021) 3:186–95. doi: 10.52223/jei3032108

[101] Stewart WF Ricci JA Chee E Hahn SR Morganstein D. Cost of lost productive work time among US workers with depression. JAMA. (2003) 289:3135–44. doi: 10.1001/jama.289.23.3135 12813119

[102] White MP Elliott LR Taylor T Wheeler BW Spencer A . Recreational physical activity in natural environments and implications for health: a population based cross-sectional study in England. Prev Med. (2016) 91:383–8. doi: 10.1016/j.ypmed.2016.08.023 27658650

[103] Carty E. Outdoor adventure youth work: bridging child and youth care and outdoor adventure. In: Child & Youth Care Across Sectors: Canadian Perspectives. Toronto: Canadian Scholars Press (2007).

[104] Vespestad M Mehmetoglu M. The relationship between tourist nationality, cultural orientation and nature-based tourism experiences. Eur J Tour Res. (2010) 3:87–104. doi: 10.54055/ejtr.v3i2.50

[105] Irvine KN Fisher D Marselle MR Currie M Colley K Warber SL. Social Isolation in older adults: a qualitative study on the social dimensions of group outdoor health walks. Int J Environ Res Public Health. (2022) 19:5353. doi: 10.3390/ijerph19095353 35564752 PMC9103571

[106] Rigby BP Dodd-Reynolds CJ Oliver EJ. Inequities and inequalities in outdoor walking groups: a scoping review. Public Health Rev. (2020) 41:4. doi: 10.1186/s40985-020-00119-4 32190410 PMC7071574

[107] Hamel E. Wild Civility: Cultivating the Foundations of Social Justice Through Participation in a Wilderness Program. Saarbrücken: LAP LAMBERT Academic Publishing (2012).

[108] Wilcer S. Understanding hiking participation and benefits: lessons learned from the first day hikes initiative (thesis). Clemson: Clemson University (2017).

[109] Kotut L Horning M Haqq D Niu S Stelter T McCrickard DS . Tensions on trails: understanding differences between group and community needs in outdoor settings. arXiv [Preprint]. arXiv:1810.08666. (2018). Available online at: http://arxiv.org/abs/1810.08666

[110] Ziółkowska J. Finding opportunities in uncertain times. The case study of a tourist guides venture in the EU. Sustainability. (2021) 13:12959. doi: 10.3390/su132312959

[111] Nilsson J Zillinger M. There is No Such Thing as a Free Walk: Spatial Implications of Shared Guiding Developments. Östersund: Mid Sweden University (2018). Available online at: https://www.semanticscholar.org/paper/There-is-no-such-thing-as-a-free-walk%3A-Spatial-of-Nilsson-Zillinger/7f2894d6cc32531b0f331341262c1c2706eca1f4

[112] Bruno B Faggini M. Sharing competition: an agent-based model for the short-term accommodations market. B.E. J Econ Anal Policy. (2020) 20:1–13. doi: 10.1515/bejeap-2019-0231

[113] Zervas G Proserpio D Byers JW. The rise of the sharing economy: estimating the impact of airbnb on the hotel industry. J Market Res. (2017) 54:687–705. doi: 10.1509/jmr.15.0204

[114] Guttentag D. Airbnb: disruptive innovation and the rise of an informal tourism accommodation sector. Curr Issues Tour. (2015) 18:1–26. doi: 10.1080/13683500.2013.827159

[115] Chakraborty A Hannak A Biega AJ Gummadi KL. Fair sharing for sharing economy platforms. In: Proceedings of FATREC Workshop on Responsible Recommendation at RecSys, Como, Italy (2017). Boise, ID: Boise State University. Available online at: http://scholarworks.boisestate.edu/fatrec/2017/1/6

[116] Gössling S Hall CM. Sharing versus collaborative economy: how to align ICT developments and the SDGs in tourism? J Sustain Tour. (2019) 27:74–96. doi: 10.1080/09669582.2018.1560455

[117] Stanley P. Unlikely hikers? Activism, Instagram, and the queer mobilities of fat hikers, women hiking alone, and hikers of colour. Mobilities. (2020) 15:241–56. doi: 10.1080/17450101.2019.1696038

[118] Winter PL Crano WD Lamb CS Basáñez T. Equity in access to outdoor recreation—informing a sustainable future. Sustainability. (2020) 12:124. doi: 10.3390/su12010124

[119] Rödl M Haider J Joosse S. The quest for “nature” in selfies: how platforms shape nature/society relationships. J Environ Plan Manag. (2024) 67:1928–51. doi: 10.1080/09640568.2023.2265548

[120] D'Hooghe S Inaç Y Deforche B Dyck DV de Ridder K Vandevijvere S . The role of the perceived environment for recreational walking among adults in socioeconomically disadvantaged situations: a study using walk-along interviews. SSM Popul Health. (2023) 23:101456. doi: 10.1016/j.ssmph.2023.101456 37501782 PMC10368917

[121] Lindell SK. Reconciling technology and nature : the use of mobile technology in outdoor recreation (thesis). Western Washington University, Bellingham, WA, United States (2014). Available online at: https://cedar.wwu.edu/wwuet/346

[122] Hartfiel N Gittins H Morrison V Wynne-Jones S Dandy N Edwards RT. Social return on investment of nature-based activities for adults with mental wellbeing challenges. Int J Environ Res Public Health. (2023) 20:6500. doi: 10.3390/ijerph20156500 37569040 PMC10418598

[123] Buckley R C Brough P. Economic value of parks via human mental health: an analytical framework. Front Ecol Evol. (2017) 5. doi: 10.3389/fevo.2017.00016

[124] Buckley R Brough P Hague L Chauvenet A Fleming C Roche E . Economic value of protected areas via visitor mental health. Nat Commun. (2019) 10:5005. doi: 10.1038/s41467-019-12631-6 31719526 PMC6851373

[125] Busbee RL. Maximizing economic benefits from a rails-to-trails project in southern West Virginia: a case study of the greenbriar river trail. Huntington, WV: Marshall University (2001). Available online at: https://trid.trb.org/View/585109.

[126] Li Z Mao L. Construction of a national trail research framework under a natural protected area system. Sustainability. (2022) 14:12343. doi: 10.3390/su141912343

[127] Moore Roger L Barthlow K. North Carolina State University. The Economic Impacts and Uses of Long-Distance Trails: Featuring a Case Study of the Overmountain Victory National Historic Trail. Raleigh, NC: North Carolina State University, Department of Parks, Recreation and Tourism Management (1998).

[128] Slabbert LM. The Impact of an Accreditation System for Trails on Growth in Hiking Tourism - ProQuest. University of Pretoria, Pretoria, South Africa (2015). Available online at: https://www.proquest.com/openview/344f33432b56cebd66e72d762348bb22/1?pq-origsite=gscholar&cbl=2026366&diss=y

[129] Jarolímková L Vaníček J Bejdáková B. Evaluation of the benefits of the certification leading quality trails – best of Europe: case study LuŽnice Valley Hiking Trail. In: Tourism in Southern and Eastern Europe 2021: ToSEE – Smart, Experience, Excellence & ToFEEL – Feelings, Excitement, Education, Leisure (2021). p. 375–85. doi: 10.20867/tosee.06.25

[130] Liu Y Salbach NM Webber SC Barclay R. Individual and environmental variables related to outdoor walking among older adults: verifying a model to guide the design of interventions targeting outdoor walking. PLoS ONE. (2024) 19:e0296216. doi: 10.1371/journal.pone.0296216 38198462 PMC10781134

[131] Salbach NM Barclay R Webber SC Jones CA Mayo NE Lix LM . A theory-based, task-oriented, outdoor walking programme for older adults with difficulty walking outdoors: protocol for the Getting Older Adults Outdoors (GO-OUT) randomised controlled trial. BMJ Open. (2019) 9:e029393. doi: 10.1136/bmjopen-2019-029393 31005945 PMC6500266

[132] Vance LK Jones GP Lemly JM Newlon KR Colorado Natural Heritage Program Montana Natural Heritage Program . Assessing the Natural Range of Variability in Minimally Disturbed Wetlands Across the Rocky Mountains : The Rocky Mountain ReMAP Project. Helena, MT: Montana Natural Heritage Program (2012). doi: 10.5962/bhl.title.65647

[133] Huffman M G. Trouble in paradise - accident trends in the outdoors. In: Proceedings of the 1998 International Conference on Outdoor Recreation. Salt Lake City: University of Utah Press (1999).

[134] Wachira L Muthomi H Ooko W. Outdoor adventure practice in Kenya: injuries, illnesses, non-medical concerns, and evacuation profiles on Mt. Kenya. J Human Perf Extrem Environ. (2021) 16. doi: 10.7771/2327-2937.1137

[135] Sahri S Raharjo BB Nasuka N Sumartiningsih S Kresnajati S Sugiarto S . Criticality of preparation and equipment in hiking and trekking activities: a systematic review. Retos. (2024) 61:210–7. doi: 10.47197/retos.v61.108220

[136] Manca A Meloni M Morrone M Boi A Martinez G Ventura L . Wild trekking as an opportunity for rapidly improving anthropometrics, cardiorespiratory and muscular performance in active older adults: the Sardinia “Selvaggio Blu” experience. Sport Sci Health. (2024) 20:609–18. doi: 10.1007/s11332-023-01150-z

[137] Jing D Zheng Y Jialong R Jianing C . The systematic construction of emergency medical security system in long-distance walking movement on urban paved roads. Int J Med Sci Clin Res Stud. (2024) 04. doi: 10.47191/ijmscrs/v4-i03-18

[138] Freeman EL. Walking through and being with nature : an examination of meaning-making and human-environment interaction in two walking and solo experiences in UK wild places (thesis). University of Leeds, Leeds, England (2013).

[139] Wu Y Wei YD Liu M García I. Green infrastructure inequality in the context of COVID-19: taking parks and trails as examples. Urban For Urban Green. (2023) 86:128027. doi: 10.1016/j.ufug.2023.128027 37614701 PMC10443926

[140] Reed JA Ballard RM Hill M Berrigan D. Identification of effective programs to improve access to and use of trails among youth from under-resourced communities: a review. Int J Environ Res Public Health. (2020) 17:7707. doi: 10.3390/ijerph17217707 33105592 PMC7659949

[141] Strife S Downey L. Childhood development and access to nature. Organ Environ. (2009) 22:99–122. doi: 10.1177/1086026609333340 21874103 PMC3162362

[142] Werner A. Key challenges facing the development of accessible tourism, using the example of Szczecin in Poland. Eur Res Stud J. (2023) 26:680–9. doi: 10.35808/ersj/3241

[143] Reece LJ Quirk H Wellington C Haake SJ Wilson F. Bright Spots, physical activity investments that work: Parkrun; a global initiative striving for healthier and happier communities. Br J Sports Med. (2019) 53:326–7. doi: 10.1136/bjsports-2018-100041 30262451

[144] Schneider PP Smith RA Bullas AM Quirk H Bayley T Haake SJ . Multiple deprivation and geographic distance to community physical activity events—achieving equitable access to parkrun in England. Public Health. (2020) 189: 48–53. doi: 10.1016/j.puhe.2020.09.002 PMC7762722 33157459

[145] Rigolon A Flohr TL. Access to Parks for youth as an environmental justice issue: access inequalities and possible solutions. Buildings. (2014) 4:69–94. doi: 10.3390/buildings4020069

[146] Hanson S. Towards an understanding of walking groups as a health promoting intervention (thesis). University of East Anglia, Norwich Medical School, Norwich, England (2016). Available online at: https://ueaeprints.uea.ac.uk/id/eprint/59466/

[147] Salvo D Garcia L Reis RS Stankov I Goel R Schipperijin J . Physical activity promotion and the United Nations sustainable development goals: building synergies to maximize impact. J Phys Act Health. (2021). 18:1163–80. doi: 10.1123/jpah.2021-0413 34257157

[148] Malchrowicz-Mośko E Botiková Z Poczta J. “Because we don't want to run in smog”: problems with the sustainable management of sport event tourism in protected areas (a case study of National Parks in Poland and Slovakia). Sustainability. (2019) 11:325. doi: 10.3390/su11020325

[149] Boonyasurat PY Jongwanich JW. Eco-trekking in Southeast Asia: a comparative study of sustainable practices in mountain tourism. J Hosp Tour Manag. (2023) 6:12–22. doi: 10.53819/81018102t4175

[150] Green RJ Croft DB Wolf ID. Preface: special issue on environmental impact of nature-based tourism. Environments. (2019) 6:112. doi: 10.3390/environments6100112

[151] Leung Y Marion J. Recreation Impacts and Management in Wilderness: A State-of-Knowledge Review. Ogden, UT: USDA Forest Service, Rocky Mountain Research Station (2000).

[152] Barros A Marina Pickering C. How networks of informal trails cause landscape level damage to vegetation. Environ Manage. (2017) 60:57–68. doi: 10.1007/s00267-017-0865-9 28412764

[153] Gathoni B Rintaugu EG Munayi SP. Effectiveness of the management measures undertaken to mitigate the impact of recreational activities on vegetation, soil, water and wild game. Int J Sociol. (2022) 6:11–9.

[154] Taylor AR Knight RL. Wildlife responses to recreation and associated visitor perceptions. Ecol Appl. (2025) 13:951–63. doi: 10.1890/1051-0761(2003)13[951:WRTRAA]2.0.CO;2

[155] Pickering CM Hill W. Impacts of recreation and tourism on plant biodiversity and vegetation in protected areas in Australia. J Environ Manag. (2007) 85:791–800. doi: 10.1016/j.jenvman.2006.11.021 17234325

[156] Wójcicki A Switlik W Dobrowolska D. The need for wildlife research and improved management of protected areas in the face of increased nature-based tourism. Environ Prot Nat Resour. (2023) 34:20–8. doi: 10.2478/oszn-2023-0009

[157] Benner M. The decline of tourist destinations: an evolutionary perspective on overtourism. Sustainability. (2020) 12:3653. doi: 10.3390/su12093653

[158] Kuščer K Mihalič T. Residents' attitudes towards overtourism from the perspective of tourism impacts and cooperation—the case of Ljubljana. Sustainability. (2019) 11:1823. doi: 10.3390/su11061823

[159] Taiminen S. The negative impacts of overtourism on tourism destination from environmental and socio-cultural perspectives (thesis). Yrkeshögskolan Arcada, Helsinki, Finland (2018). Available online at: http://www.theseus.fi/handle/10024/158561

[160] Thomas SL Reed SE. Entrenched ties between outdoor recreation and conservation pose challenges for sustainable land management. Environ Res Lett. (2019) 14:115009. doi: 10.1088/1748-9326/ab4f52

[161] Randall A. A difficulty with the travel cost method. Land Econ. (1994) 70:88–96. doi: 10.2307/3146443

[162] Goff R. The economic value of tourism and recreation in forested areas of Western Australia (thesis). Edith Cowan University, Joondalup, Australia (2003). Available online at: https://ro.ecu.edu.au/theses/1302

[163] Fix P Loomis J. The economic benefits of mountain biking at one of its meccas: an application of the travel cost method to mountain biking in Moab, Utah. J Leisure Res. (1997) 29:342–52. doi: 10.1080/00222216.1997.11949800

[164] Ormerod NS. An Examination of the Challenges of Capturing the Value of Adventurous Off-Road Cycling: A Perspective from South West England – ProQuest (2025). Available online at: https://www.proquest.com/openview/3fdc335a98ae3456b9b2d9852827c346/1?pq-origsite=gscholar&cbl=51922&diss=y (Accessed October 20, 2025).

[165] Manning R Parks Stewardship Forum. Walking the Talk in America's National Parks – ProQuest (2025). Available online at: https://www.proquest.com/openview/c56ad46a98746abb4fa8f962776aa862/1?pq-origsite=gscholar&cbl=55002 (Accessed October 20, 2025).

[166] Hartig T Mitchell R Vries S de Frumkin H. Nature and health. Ann Rev Public Health. (2014) 35:207–28. doi: 10.1146/annurev-publhealth-032013-182443 24387090

[167] Anzman-Frasca S Drozdowsky J Zayatz C Holmbeck K. Effects of a randomized controlled hiking intervention on daily activities, sleep, and stress among adults during the COVID-19 pandemic. BMC Public Health. (2023) 23:892. doi: 10.1186/s12889-023-15696-7 37189100 PMC10184062

[168] Bowler DE Buyung-Ali LM Knight TM Pullin AS. A systematic review of evidence for the added benefits to health of exposure to natural environments. BMC Public Health. (2010) 10:456. doi: 10.1186/1471-2458-10-456 20684754 PMC2924288

[169] Holland WH Powell RB Thomsen JM Monz CA. A systematic review of the psychological, social, and educational outcomes associated with participation in wildland recreational activities. J Outdoor Recreat Educ Leadership. (2018) 10:197–225. doi: 10.18666/JOREL-2018-V10-I3-8382

[170] Kwan RYC Salihu D Lee PH Tse M Cheung DSK Roopsawang I . The effect of e-health interventions promoting physical activity in older people: a systematic review and meta-analysis. Eur Rev Aging Phys Act. (2020) 17:7. doi: 10.1186/s11556-020-00239-5 32336996 PMC7175509

[171] Meredith GR Rakow DA Eldermire ERB Madsen CG Shelley SP . Minimum time dose in nature to positively impact the mental health of college-aged students, and how to measure it: a scoping review. Front Psychol. (2020) 10:2942. doi: 10.3389/fpsyg.2019.02942 31993007 PMC6970969

[172] Struthers NA Guluzade NA Zecevic AA Walton DM Gunz A. Nature-based interventions for physical health conditions: a systematic review and meta-analysis. Environ Res. (2024) 258:119421. doi: 10.1016/j.envres.2024.119421 38876421

[173] Lukoseviciute G Pereira LN Panagopoulos T Fedeli G Ramsey E Madden K . Recreational trail development within different geographical contexts as a determinant of income multiplier and local economic impact. Tour Manag Perspect. (2023) 46:101090. doi: 10.1016/j.tmp.2023.101090

[174] Cummings JP. A Longitudinal Study of the Outcomes from Participation in Wilderness Adventure Education Programs – ProQuest (2025). Available online at: https://www.proquest.com/openview/f8e6a153d6fc2e1a7f5fdb876347ae7d/1?pq-origsite=gscholar&cbl=18750&diss=y (Accessed October 20, 2025).

[175] Chhetri P Arrowsmith C Jackson M. Determining hiking experiences in nature-based tourist destinations. Tour Manag. (2004) 25:31–43. doi: 10.1016/S0261-5177(03)00057-8

[176] Smith RA Schneider PP Cosulich R Quirk H Bullas AM Haake SJ . Socioeconomic inequalities in distance to and participation in a community-based running and walking activity: a longitudinal ecological study of parkrun 2010 to 2019. Health Place. (2021) 71:102626. doi: 10.1016/j.healthplace.2021.102626 34333371 PMC8522482

[177] Reuter S Kemmerling T Schmalenbach T Brözel NC. Economic impacts of trail destinations: the case of the peaks of the Balkans trail. J Outdoor Recreat Tour. (2025) 52:100928. doi: 10.1016/j.jort.2025.100928

