# Supplementary material for: An integrative review of the physical, mental, and socioeconomic benefits of outdoor hiking
Source: Front Public Health. 2026 Jan 7;13:1700325. doi: 10.3389/fpubh.2025.1700325 (PMC12819716; doi:10.3389/fpubh.2025.1700325)
Supplement: Supplementary file 1 [file Table_1.docx]

Supplementary Material

# Supplementary Table S1. Search Strategies for PubMed, Scopus, Web of Science，PsycINFO and SPORTDiscus

| ****Database**** | **Search Strategy** |
| --- | --- |
| PubMed | (("Hiking"[Mesh] OR hiking[tiab] OR trek*[tiab] OR "trail walking"[tiab] OR "Outdoor Recreation"[Mesh] OR "outdoor walking"[tiab] OR "nature walk*"[tiab] OR "mountain walking"[tiab] OR "nature-based physical activit*"[tiab]))  AND  (("Physical Fitness"[Mesh] OR "Exercise"[Mesh] OR "Health"[Mesh] OR "Physical Activity"[Mesh] OR "Physical Health"[tiab] OR "Cardiovascular Health"[tiab] OR "Immune Function"[tiab] OR "Body Composition"[tiab] OR "Metabolic Health"[tiab]  OR "Mental Health"[Mesh] OR "Psychological Well-Being"[tiab] OR wellbeing[tiab] OR "Depression"[Mesh] OR "Anxiety"[Mesh] OR "Mood"[tiab] OR "Quality of Life"[Mesh])  OR  ("Economics"[Mesh] OR "Cost-Benefit Analysis"[Mesh] OR "Health Economics"[tiab] OR "Economic Impact"[tiab] OR "Tourism"[Mesh] OR "Community Development"[tiab] OR "Regional Economy"[tiab] OR "Social Cohesion"[tiab] OR "Socioeconomic Factors"[Mesh] OR "Environmental Conservation"[tiab])) |
| Scopus | (TITLE-ABS-KEY (hiking OR trek* OR "trail walking" OR "outdoor walking" OR "nature walk*" OR "mountain walking" OR "nature-based physical activit*"))  AND  (TITLE-ABS-KEY ("physical health" OR "mental health" OR wellbeing OR "psychological well-being" OR depression OR anxiety OR stress OR "quality of life" OR cardiovascular OR metabolic OR "immune function" OR "body composition")  OR  TITLE-ABS-KEY ("economic impact" OR "cost-benefit" OR "health economics" OR tourism OR "community development" OR "regional economy" OR "social cohesion" OR "socioeconomic factors" OR "environmental protection")) |
| Web of Science | TS = ((hiking OR trek* OR "trail walking" OR "outdoor walking" OR "nature walk*" OR "mountain walking" OR "nature-based physical activit*") AND (("physical health" OR "mental health" OR wellbeing OR "psychological well-being" OR depression OR anxiety OR stress OR "quality of life" OR cardiovascular OR metabolic OR "immune function" OR "body composition") OR ("economic impact" OR "cost-benefit" OR "health economics" OR tourism OR "community development" OR "regional economy" OR "social cohesion" OR "socioeconomic factors" OR "environmental protection"))) |
| PsycINFO and SPORTDiscus (EBSCOhost platform) | ( ( (MH "Hiking" OR MH "Trekking" OR MH "Backpacking" OR MH "Trail Walking" OR MH "Mountain Walking" OR MH "Outdoor Recreation" OR MH "Nature-Based Recreation") OR ("hiking" OR "trekking" OR "trail walking" OR "mountain walking" OR "walking in nature" OR "nature-based walking" OR "outdoor walking" OR "nature trail*" OR "eco-hiking" OR "green exercise").ti,ab. ) AND ( (MH "Mental Health" OR MH "Psychological Wellbeing" OR MH "Stress" OR MH "Depression" OR MH "Anxiety" OR MH "Well Being" OR MH "Social Interaction" OR MH "Community Participation" OR MH "Economic Impact") OR ("mental health" OR "psychological wellbeing" OR "emotional wellbeing" OR "stress reduction" OR "depression" OR "anxiety" OR "mood" OR "happiness" OR "life satisfaction" OR "social cohesion" OR "community engagement" OR "economic benefit" OR "wellbeing economy").ti,ab. )) |


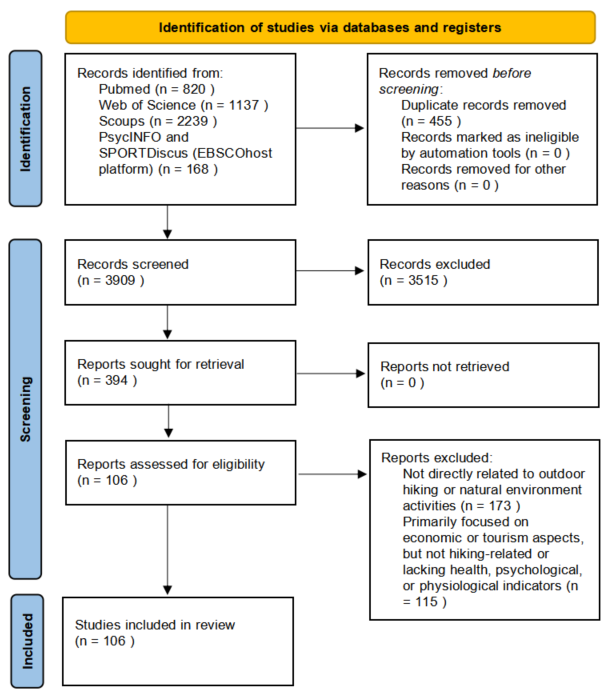


**Supplementary Figure S1. PRISMA flow diagram.**

# Supplementary Table S2. AMSTAR 2 items for Oja, P.(2018)

| **Domain number** | **Critical or non-critical** | **Content of the domain** | **Yes / Partial yes / No** |
| --- | --- | --- | --- |
| 1 | Non-critical domain | Did the research questions and inclusion criteria for the review include the components of PICOa? | **Yes** |
| 2 | Critical domain | Did the report of the review contain an explicit statement that the review methods were established prior to the conduct of the review and did the report justify any significant deviations from the protocol? | **No** |
| 3 | Non-critical domain | Did the review authors explain their selection of the study designs for inclusion in the review? | **Yes** |
| 4 | Critical domain | Did the review authors use a comprehensive literature search strategy? | **Partial yes** |
| 5 | Non-critical domain | Did the review authors perform study selection in duplicate? | **Yes** |
| 6 | Non-critical domain | Did the review authors perform data extraction in duplicate? | **Yes** |
| 7 | Critical domain | Did the review authors provide a list of excluded studies and justify the exclusions? | **No** |
| 8 | Non-critical domain | Did the review authors describe the included studies in adequate detail? | **Yes** |
| 9 | Critical domain | Did the review authors use a satisfactory technique for assessing the risk of bias in individual studies that were included in the review? | **Yes** |
| 10 | Non-critical domain | Did the review authors report on the sources of funding for the studies included in the review? | **No** |
| 11 | Critical domain | If meta-analysis was performed did the review authors use appropriate methods for statistical combination of results? | **Yes** |
| 12 | Non-critical domain | If meta-analysis was performed, did the review authors assess the potential impact of risk of bias in individual studies on the results of the meta-analysis or other evidence synthesis? | **Partial yes** |
| 13 | Critical domain | Did the review authors account for risk of bias in individual studies when interpreting/ discussing the results of the review? | **Partial yes** |
| 14 | Non-critical domain | Did the review authors provide a satisfactory explanation for, and discussion of, any heterogeneity observed in the results of the review? | **No** |
| 15 | Critical domain | If they performed quantitative synthesis did the review authors carry out an adequate investigation of publication bias (small study bias) and discuss its likely impact on the results of the review? | **Yes** |
| 16 | Non-critical domain | Did the review authors report any potential sources of conflict of interest, including any funding they received for conducting the review? | **Partial yes** |

# Supplementary Table S3. AMSTAR 2 items for Coventry, P. A.(2021)

| **Domain number** | **Critical or non-critical** | **Content of the domain** | **Yes / Partial yes / No** |
| --- | --- | --- | --- |
| 1 | Non-critical domain | Did the research questions and inclusion criteria for the review include the components of PICOa? | **Yes** |
| 2 | Critical domain | Did the report of the review contain an explicit statement that the review methods were established prior to the conduct of the review and did the report justify any significant deviations from the protocol? | **Yes** |
| 3 | Non-critical domain | Did the review authors explain their selection of the study designs for inclusion in the review? | **Yes** |
| 4 | Critical domain | Did the review authors use a comprehensive literature search strategy? | **Partial yes** |
| 5 | Non-critical domain | Did the review authors perform study selection in duplicate? | **Yes** |
| 6 | Non-critical domain | Did the review authors perform data extraction in duplicate? | **Partial yes** |
| 7 | Critical domain | Did the review authors provide a list of excluded studies and justify the exclusions? | **No** |
| 8 | Non-critical domain | Did the review authors describe the included studies in adequate detail? | **Yes** |
| 9 | Critical domain | Did the review authors use a satisfactory technique for assessing the risk of bias in individual studies that were included in the review? | **Yes** |
| 10 | Non-critical domain | Did the review authors report on the sources of funding for the studies included in the review? | **No** |
| 11 | Critical domain | If meta-analysis was performed did the review authors use appropriate methods for statistical combination of results? | **Yes** |
| 12 | Non-critical domain | If meta-analysis was performed, did the review authors assess the potential impact of risk of bias in individual studies on the results of the meta-analysis or other evidence synthesis? | **Partial yes** |
| 13 | Critical domain | Did the review authors account for risk of bias in individual studies when interpreting/ discussing the results of the review? | **Yes** |
| 14 | Non-critical domain | Did the review authors provide a satisfactory explanation for, and discussion of, any heterogeneity observed in the results of the review? | **Yes** |
| 15 | Critical domain | If they performed quantitative synthesis did the review authors carry out an adequate investigation of publication bias (small study bias) and discuss its likely impact on the results of the review? | **Partial yes** |
| 16 | Non-critical domain | Did the review authors report any potential sources of conflict of interest, including any funding they received for conducting the review? | **Yes** |

# Supplementary Table S4. AMSTAR 2 items for Hanson, S., & Jones, A. (2015)

| **Domain number** | **Critical or non-critical** | **Content of the domain** | **Yes / Partial yes / No** |
| --- | --- | --- | --- |
| 1 | Non-critical domain | Did the research questions and inclusion criteria for the review include the components of PICOa? | **Yes** |
| 2 | Critical domain | Did the report of the review contain an explicit statement that the review methods were established prior to the conduct of the review and did the report justify any significant deviations from the protocol? | **Yes** |
| 3 | Non-critical domain | Did the review authors explain their selection of the study designs for inclusion in the review? | **Partial yes** |
| 4 | Critical domain | Did the review authors use a comprehensive literature search strategy? | **Yes** |
| 5 | Non-critical domain | Did the review authors perform study selection in duplicate? | **Partial yes** |
| 6 | Non-critical domain | Did the review authors perform data extraction in duplicate? | **No** |
| 7 | Critical domain | Did the review authors provide a list of excluded studies and justify the exclusions? | **No** |
| 8 | Non-critical domain | Did the review authors describe the included studies in adequate detail? | **Yes** |
| 9 | Critical domain | Did the review authors use a satisfactory technique for assessing the risk of bias in individual studies that were included in the review? | **Partial yes** |
| 10 | Non-critical domain | Did the review authors report on the sources of funding for the studies included in the review? | **No** |
| 11 | Critical domain | If meta-analysis was performed did the review authors use appropriate methods for statistical combination of results? | **Yes** |
| 12 | Non-critical domain | If meta-analysis was performed, did the review authors assess the potential impact of risk of bias in individual studies on the results of the meta-analysis or other evidence synthesis? | **Partial yes** |
| 13 | Critical domain | Did the review authors account for risk of bias in individual studies when interpreting/ discussing the results of the review? | **Partial yes** |
| 14 | Non-critical domain | Did the review authors provide a satisfactory explanation for, and discussion of, any heterogeneity observed in the results of the review? | **No** |
| 15 | Critical domain | If they performed quantitative synthesis did the review authors carry out an adequate investigation of publication bias (small study bias) and discuss its likely impact on the results of the review? | **Yes** |
| 16 | Non-critical domain | Did the review authors report any potential sources of conflict of interest, including any funding they received for conducting the review? | **Yes** |

# Supplementary Table S5. AMSTAR 2 items for Grassini S. (2022)

| **Domain number** | **Critical or non-critical** | **Content of the domain** | **Yes / Partial yes / No** |
| --- | --- | --- | --- |
| 1 | Non-critical domain | Did the research questions and inclusion criteria for the review include the components of PICOa? | **Yes** |
| 2 | Critical domain | Did the report of the review contain an explicit statement that the review methods were established prior to the conduct of the review and did the report justify any significant deviations from the protocol? | **No** |
| 3 | Non-critical domain | Did the review authors explain their selection of the study designs for inclusion in the review? | **Partial yes** |
| 4 | Critical domain | Did the review authors use a comprehensive literature search strategy? | **Partial yes** |
| 5 | Non-critical domain | Did the review authors perform study selection in duplicate? | **No** |
| 6 | Non-critical domain | Did the review authors perform data extraction in duplicate? | **No** |
| 7 | Critical domain | Did the review authors provide a list of excluded studies and justify the exclusions? | **No** |
| 8 | Non-critical domain | Did the review authors describe the included studies in adequate detail? | **Partial yes** |
| 9 | Critical domain | Did the review authors use a satisfactory technique for assessing the risk of bias in individual studies that were included in the review? | **Yes** |
| 10 | Non-critical domain | Did the review authors report on the sources of funding for the studies included in the review? | **No** |
| 11 | Critical domain | If meta-analysis was performed did the review authors use appropriate methods for statistical combination of results? | **Yes** |
| 12 | Non-critical domain | If meta-analysis was performed, did the review authors assess the potential impact of risk of bias in individual studies on the results of the meta-analysis or other evidence synthesis? | **Partial yes** |
| 13 | Critical domain | Did the review authors account for risk of bias in individual studies when interpreting/ discussing the results of the review? | **Partial yes** |
| 14 | Non-critical domain | Did the review authors provide a satisfactory explanation for, and discussion of, any heterogeneity observed in the results of the review? | **Partial yes** |
| 15 | Critical domain | If they performed quantitative synthesis did the review authors carry out an adequate investigation of publication bias (small study bias) and discuss its likely impact on the results of the review? | **Partial yes** |
| 16 | Non-critical domain | Did the review authors report any potential sources of conflict of interest, including any funding they received for conducting the review? | **Yes** |

Note: Yes : Fully meets the criteria; Partly yes : Partially meets the criteria but with important limitations; No : Does not meet the criteria or fails to report relevant information. Items 2, 4, 7, 9, 11, 13 and 15 are critical items for deriving the confidence rating in the results of the SR. Abbreviations: AMSTAR 2, A Measurement Tool to Assess Systematic Reviews, Version 2; MA, meta-analysis; PICO, Population, Intervention, Comparison, Outcome; RoB, risk of bias; SR, systematic review.

| **Supplementary Table S5. Summary of Literature Screening** | |
| --- | --- |
| **Physical and psychological health** | |
| **Author (Year)** | **Title** |
| ROMAIN (2019) | Association between physical multimorbidity, body mass index and mental health |
| LAVALLEE (2021) | Obesity and Mental Health: A Longitudinal, Cross-Cultural Examination in Germany and China |
| GEIGER (2023) | Needs and Demands for e-Mental Health Interventions in Individuals with Overweight and Obesity: User-Centred Design Approach |
| MURO (2023) | Forest bathing and hiking benefits for mental health during the COVID-19 pandemic in Mediterranean regions |
| TEIXEIRA (2021) | Obesity and Natural Spaces in Adults and Older People: A Systematic Review |
| FATTORINI (2012) | Workload Comparison Between Hiking and Indoor Physical Activity |
| ACEVEDO-DUQUE Á (2022) | Scientometric Analysis of Hiking Tourism and Its Relevance for Wellbeing and Knowledge Management |
| MITTEN (2018) | Hiking: A Low-Cost, Accessible Intervention to Promote Health Benefits] |
| LIAO X (2019) | Maternal manganese activates anti-apoptotic-related gene expressions via miR-1551 and miR-34c in embryonic hearts from maternal heat stress (Gallus gallus) |
| LESSER (2025) | A Pre-Post Study Design Exploring the Potential Benefits of a Hiking Intervention for Active and Inactive Older Adults |
| NIEDERMEIER (2017) | Affective responses in mountain hiking-A randomized crossover trial focusing on differences between indoor and outdoor activity |
| XU P (2022) | Relationship between physical activity and mental health in a national representative cross-section study: Its variations according to obesity and comorbidity |
| BETTMANN (2024) | How Does Nature Exposure Affect Adults With Symptoms of Mental Illness? A Meta-Analysis |
| SMILEY (2020) | Comparing the Trail Users with Trail Non-Users on Physical Activity, Sleep, Mood and Well-Being Index |
| HUBER (2023) | Long-Term Effects of Mountain Hiking vs. Forest Therapy on Physical and Mental Health of Couples: A Randomized Controlled Trial |
| MALEM (2024) | Brisk Walking Exercise Has Benefits of Lowering Blood Pressure in Hypertension Sufferers: A Systematic Review and Meta-Analysis |
| WEN (2011) | Minimum amount of physical activity for reduced mortality and extended life expectancy: a prospective cohort study |
| SMITH (2017) | The Influence of a Cognitive Dual Task on the Gait Parameters of Healthy Older Adults: A Systematic Review and Meta-Analysis |
| MURTAGH (2010) | Walking: the first steps in cardiovascular disease prevention |
| PAPALE (2025) | The Impact of a Multidimensional Physical Activity Intervention on Glycemic Control in Type 1 Diabetes: A Preliminary Study |
| WEBB (2016) | Evaluation of cardiovascular risk-lowering health benefits accruing from laboratory-based, community-based and exercise-referral exercise programmes |
| PAPALE (2023) | Psychophysiological Data Harmonization for the Sustainability of Outdoor Activities |
| OJA P (2018) | Effects of frequency, intensity, duration and volume of walking interventions on CVD risk factors: a systematic review and meta-regression analysis of randomised controlled trials among inactive healthy adults |
| HANSON (2015) | Is there evidence that walking groups have health benefits? A systematic review and meta-analysis |
| SKALIY (2023) | Assessment of the functional state of the cardiovascular system of students during a mountain hiking trip |
| MIEDA (2021) | Education program for prevention of outdoor accidents in middle-high aged trekkers: Monitoring of change in blood pressure and heart rate during exercise |
| FAULKNER (2012) | Self-Paced Walking within a Diverse Topographical Environment Elicits an Appropriate Training Stimulus for Cardiac Rehabilitation Patients |
| MANNING (2015) | Cardiovascular and Perceived Exertion Responses to Leisure Trail Hiking |
| MARTINAITIENĖ D (2024) | A randomised controlled trial assessing the effects of weather sensitivity profile and walking in nature on the psychophysiological response to stress in individuals with coronary artery disease. A study protoco |
| LÓPEZ-POUSA S (2015) | Sense of Well-Being in Patients with Fibromyalgia: Aerobic Exercise Program in a Mature Forest-A Pilot Study |
| SHIN (2024) | Effects of Exercise Intensity Differences in Forest Therapy Programs on Immunoglobulin A and Dehydroepiandrosterone Levels in Older Adults |
| NIEDERMEIER (2017) | A Randomized Crossover Trial on Acute Stress-Related Physiological Responses to Mountain Hiking |
| FARROW (2019) | A Review of Field Experiments on the Effect of Forest Bathing on Anxiety and Heart Rate Variability |
| MAU (2021) | Are Long-Distance Walks Therapeutic? A Systematic Scoping Review of the Conceptualization of Long-Distance Walking and Its Relation to Mental Health |
| SUDIMAC (2022) | How nature nurtures: Amygdala activity decreases as the result of a one-hour walk in nature |
| WANG (2023) | The Association of Outdoor Walking Per Week with Mental Health and Costs of Psychotropic Drugs in Adults |
| WALTER (2019) | Comparison of surf and hike therapy for active duty service members with major depressive disorder: Study protocol for a randomized controlled trial of novel interventions in a naturalistic setting |
| THOMPSON (2011) | Does participating in physical activity in outdoor natural environments have a greater effect on physical and mental wellbeing than physical activity indoors? A systematic review |
| ROBERTS (2018) | Why Do You Ride?: A Characterization of Mountain Bikers, Their Engagement Methods, and Perceived Links to Mental Health and Well-Being |
| BARTON (2010) | What is the Best Dose of Nature and Green Exercise for Improving Mental Health? A Multi-Study Analysis |
| GRASSINI (2022) | A Systematic Review and Meta-Analysis of Nature Walk as an Intervention for Anxiety and Depression |
| COVENTRY (2021) | Nature-based outdoor activities for mental and physical health: Systematic review and meta-analysis |
| BRATMAN (2015) | Nature experience reduces rumination and subgenual prefrontal cortex activation |
| PIVA (2024) | Effects of forest walking on physical and mental health in elderly populations: a systematic review |
| NOSEWORTHY (2023) | The Effects of Outdoor versus Indoor Exercise on Psychological Health, Physical Health, and Physical Activity Behaviour: A Systematic Review of Longitudinal Trials |
| PARK (2010) | The physiological effects of Shinrin-yoku (taking in the forest atmosphere or forest bathing): evidence from field experiments in 24 forests across Japan |
| KELLY (2018) | Walking on sunshine: scoping review of the evidence for walking and mental health |
| GRECO (2024) | Impact of Coastal Walking Outdoors and Virtual Reality Indoor Walking on Heart Rate, Enjoyment Levels and Mindfulness Experiences in Healthy Adults |
| NIEDERMEIER (2019) | The Role of Anthropogenic Elements in the Environment for Affective States and Cortisol Concentration in Mountain Hiking—A Crossover Trial |
| BAILEY (2022) | Walking and Sitting Outdoors: Which Is Better for Cognitive Performance and Mental States? |
| VICTORSON (2021) | Psychosocial and Biological Outcomes of Immersive, Mindfulness-Based Treks in Nature for Groups of Young Adults and Caregivers Affected by Cancer: Results from a Single Arm Program Evaluation from 2016-2021 |
| KLEINSTÄUBER M (2017) | Rock climbing and acute emotion regulation in patients with major depressive disorder in the context of a psychological inpatient treatment: a controlled pilot trial |
| LUTTENBERGER (2015) | Indoor rock climbing (bouldering) as a new treatment for depression: study design of a waitlist-controlled randomized group pilot study and the first results |
| DORSCHT (2019) | A German climbing study on depression: a bouldering psychotherapeutic group intervention in outpatients compared with state-of-the-art cognitive behavioural group therapy and physical activation – study protocol for a multicentre randomised controlled trial |
| WALTER (2023) | A randomized controlled trial of surf and hike therapy for U.S. active duty service members with major depressive disorder |
| MELONI (2025) | Land- and water-based sports activities in natural environments as a group exercise for Parkinson’s disease: proof-of-concept pilot study] |
| MA J (2024) | Effectiveness of nature-based walking interventions in improving mental health in adults: a systematic review |
| ROSA (2023) | The Effect of Nature-Based Adventure Interventions on Depression: A Systematic Review |
| PARK (2022) | What Activities in Forests Are Beneficial for Human Health? A Systematic Review |
| LAMATUNGGA (2024) | Forests serve vulnerable groups in times of crises: improved mental health of older adults by individual forest walking during the COVID-19 pandemic |
| LEE (2025) | Influence of Forest Therapy on Cardiovascular Relaxation in Young Adults |
| LEE (2021) | Influence of Forest Visitors’ Perceived Restorativeness on Social-Psychological Stress |
| WAN (2024) | Progress and Prospects of Research on the Impact of Forest Therapy on Mental Health: A Bibliometric Analysis |
| MAYER (2021) | Motivation and mental well-being of long-distance hikers: A quantitative and qualitative approach |
| MARSELLE (2013) | Walking for well-being: are group walks in certain types of natural environments better for well-being than group walks in urban environments? |
| WEN (2023) | The Effects of Dynamic and Static Forest Bathing (Shinrin-yoku) on Physiological and Psychological Health in Males and Females |
| KANCHIBHOTLA (2022) | Alleviating Work Exhaustion, Improving Professional Fulfillment, and Influencing Positivity Among Healthcare Professionals During COVID-19: A Study on Sudarshan Kriya Yoga |
| ENRIQUE (2015) | El Mejor Self Posible: Una intervención dirigida a generar emociones positivas. Resultados preliminares |
| SONG (2022) | Restorative Effects from Green Exposure: A Systematic Review and Meta-Analysis of Randomized Control Trials |
| STOLTZFUS (2020) | Blood Pressure Changes While Hiking at Moderate Altitudes: A Prospective Cohort Study |
| KASTENHOLZ (2007) | Discussing the Potential Benefits of Hiking Tourism in Portugal |
| MANCA (2024) | Wild trekking as an opportunity for rapidly improving anthropometrics, cardiorespiratory and muscular performance in active older adults: the Sardinia “Selvaggio Blu” experience |
| HANSON (2016) | Towards an understanding of walking groups as a health promoting intervention |
| ANZMAN-FRASCA (2023) | Effects of a randomized controlled hiking intervention on daily activities, sleep, and stress among adults during the COVID-19 pandemic |
| HOLLAND (2018) | A Systematic Review of the Psychological, Social, and Educational Outcomes Associated With Participation in Wildland Recreational Activities |
| STRUTHERS (2024) | Nature-based interventions for physical health conditions: A systematic review and meta-analysis |
| **Political and economic benefits** | |
| **Author (Year)** | **Title** |
| SAMUDRA (2023) | Examining the Connection Between Health Outcomes, State Political Ideology, and Food Access in the United States |
| MAKANJUOLA (2023) | Prevention of Poor Physical and Mental Health through the Green Social Prescribing Opening Doors to the Outdoors Programme: A Social Return on Investment Analysis |
| WHITE (2016) | Federal Outdoor Recreation Trends: Effects on Economic Opportunities |
| WOODCOCK (2013) | Health impact modelling of active travel visions for England and Wales using an Integrated Transport and Health Impact Modelling Tool (ITHIM) |
| KAHLMEIER (2023) | The Health Economic Assessment Tool (HEAT) for walking and cycling - experiences from 10 years of application of a health impact assessment tool in policy and practice |
| GÖTSCHI T (2020) | Integrated Impact Assessment of Active Travel: Expanding the Scope of the Health Economic Assessment Tool (HEAT) for Walking and Cycling |
| O’MARA S (2021) | Biopsychosocial Functions of Human Walking and Adherence to Behaviourally Demanding Belief Systems: A Narrative Review |
| RAYA (2018) | Economic and social yield of investing in hiking tourism: the case of Berguedà, Spain |
| NAGLER (2013) | Community Economic Contributions from Recreational Trails Usage on Public Lands: Implications from a Comprehensive Wyoming Case Study |
| BERGSTROM (1900) | Economic Impacts of State Parks on State Economies in the South |
| ENGLISH (2025) | ECONOMIC IMPACTS OF GUIDED WHITEWATER RAFTING: A STUDY OF FIVE RIVERS1 |
| LUKOSEVICIUTE (2022) | The economic impact of recreational trails: a systematic literature review |
| COMLEY (2014) | The Economic Impact of Outdoor Recreation in the UK: The Evidence |
| DUNLOP (2025) | Economic Effect of Active Transportation Features and the Association Between the Healthcare Industry and Transportation: FHWA |
| MAPLES (2021) | Outdoor Recreation and Rural Transitions in Central Appalachia: Revisiting the Economic Impact of Rock Climbing in Kentucky’s Red River Gorge |
| IRVINE (2022) | Social Isolation in Older Adults: A Qualitative Study on the Social Dimensions of Group Outdoor Health Walks |
| KOTUT (2018) | Tensions on Trails: Understanding Differences between Group and Community Needs in Outdoor Settings |
| S D'Hooghe (2023) | The role of the perceived environment for recreational walking among adults in socioeconomically disadvantaged situations: A study using walk-along interviews |
| HARTFIEL (2023) | Social Return on Investment of Nature-Based Activities for Adults with Mental Wellbeing Challenges |
| BUCKLEY (2017) | Economic Value of Parks via Human Mental Health: An Analytical Framework |
| BUCKLEY (2019) | Economic value of protected areas via visitor mental health |
| BUSBEE (2001) | MAXIMIZING ECONOMIC BENEFITS FROM A RAILS-TO-TRAILS PROJECT IN SOUTHERN WEST VIRGINIA: A CASE STUDY OF THE GREENBRIAR RIVER TRAIL |
| MOORE (1998) | The Economic Impacts and Uses of Long-Distance Trails: Featuring a Case Study of the Overmountain Victory National Historic Trail |
| REECE (2019) | Bright Spots, physical activity investments that work: Parkrun; a global initiative striving for healthier and happier communities |
| PP Schneider (2020) | Multiple deprivation and geographic distance to community physical activity events — achieving equitable access to parkrun in England |
| GOFF (2003) | The economic value of tourism and recreation in forested areas of Western Australia |
| FIX (1997) | The Economic Benefits of Mountain Biking at One of Its Meccas: An Application of the Travel Cost Method to Mountain Biking in Moab, Utah |
| LUKOSEVICIUTE (2023) | Recreational trail development within different geographical contexts as a determinant of income multiplier and local economic impact |
| SMITH (2021) | Socioeconomic inequalities in distance to and participation in a community-based running and walking activity: A longitudinal ecological study of parkrun 2010 to 2019 |
| REUTER (2025) | Economic impacts of trail destinations: The case of the Peaks of the Balkans trail |
